# Non-Invasive Prenatal Detection of Trisomy 21 Using Tandem Single Nucleotide Polymorphisms

**DOI:** 10.1371/journal.pone.0013184

**Published:** 2010-10-08

**Authors:** Sujana Ghanta, Michael E. Mitchell, Mary Ames, Mats Hidestrand, Pippa Simpson, Mary Goetsch, William G. Thilly, Craig A. Struble, Aoy Tomita-Mitchell

**Affiliations:** 1 Division of Cardiothoracic Surgery, Department of Surgery, Medical College of Wisconsin, Milwaukee, Wisconsin, United States of America; 2 Department of Computer Science and Engineering, University of Louisville, Louisville, Kentucky, United States of America; 3 Department of Obstetrics and Gynecology, Medical College of Wisconsin, Milwaukee, Wisconsin, United States of America; 4 Division of Quantitative Health Sciences, Department of Pediatrics, Medical College of Wisconsin, Milwaukee, Wisconsin, United States of America; 5 Department of Biological Engineering, Massachusetts Institute of Technology, Cambridge, Massachusetts, United States of America; 6 Department of Mathematics, Statistics, and Computer Science, Marquette University, Milwaukee, Wisconsin, United States of America; Aga Khan University, Pakistan

## Abstract

**Background:**

Screening tests for Trisomy 21 (T21), also known as Down syndrome, are routinely performed for the majority of pregnant women. However, current tests rely on either evaluating non-specific markers, which lead to false negative and false positive results, or on invasive tests, which while highly accurate, are expensive and carry a risk of fetal loss. We outline a novel, rapid, highly sensitive, and targeted approach to non-invasively detect fetal T21 using maternal plasma DNA.

**Methods and Findings:**

Highly heterozygous tandem Single Nucleotide Polymorphism (SNP) sequences on chromosome 21 were analyzed using High-Fidelity PCR and Cycling Temperature Capillary Electrophoresis (CTCE). This approach was used to blindly analyze plasma DNA obtained from peripheral blood from 40 high risk pregnant women, in adherence to a Medical College of Wisconsin Institutional Review Board approved protocol. Tandem SNP sequences were informative when the mother was heterozygous and a third paternal haplotype was present, permitting a quantitative comparison between the maternally inherited haplotype and the paternally inherited haplotype to infer fetal chromosomal dosage by calculating a Haplotype Ratio (HR). 27 subjects were assessable; 13 subjects were not informative due to either low DNA yield or were not informative at the tandem SNP sequences examined. All results were confirmed by a procedure (amniocentesis/CVS) or at postnatal follow-up. Twenty subjects were identified as carrying a disomy 21 fetus (with two copies of chromosome 21) and seven subjects were identified as carrying a T21 fetus. The sensitivity and the specificity of the assay was 100% when HR values lying between 3/5 and 5/3 were used as a threshold for normal subjects.

**Conclusions:**

In summary, a targeted approach, based on calculation of Haplotype Ratios from tandem SNP sequences combined with a sensitive and quantitative DNA measurement technology can be used to accurately detect fetal T21 in maternal plasma when sufficient fetal DNA is present in maternal plasma.

## Introduction

Fetal chromosomal abnormalities often lead to post-birth medical conditions requiring specialized medical care that result in emotional and financial challenges to the child, parent(s) and society. Important related medical conditions can be successfully managed during the neonatal period if diagnosed early during the course of pregnancy; provided that early and accurate diagnosis exists. Conversely, these challenges increase dramatically if diagnosis is delayed [1], [2]. The most common chromosomal abnormality is Trisomy 21 (Down syndrome), which in addition to being associated with learning disabilities, is commonly associated with congenital heart disease and respiratory distress, which often require early clinical intervention to optimize clinical outcome. Because of the medical importance of early diagnosis, the American College of Obstetricians and Gynecologists (ACOG), recommends that all pregnant women, regardless of maternal age or the presence of other risk factors, be offered prenatal screening for chromosomal abnormalities (ACOG, 2007). Current non-invasive screening tools for chromosomal abnormalities typically involve a combination of ultrasound and measurement of non-specific maternal serum markers. These screening tests are limited to trisomies of chromosome 21 and 18 and are not reliable to diagnose or exclude such abnormalities. Although invasive procedures such as amniocentesis and chorionic villus sampling (CVS) are highly accurate, they carry a procedural fetal loss rate of ∼1/300 and are economically costly [3], [4].

Several methods to develop a more accurate non-invasive prenatal test for T21 have been investigated. Analysis of fetal cells in maternal circulation has been difficult due to their fragility and scarcity [5]. Analysis of free fetal nucleic acid in maternal plasma, which is present as early as five weeks gestation and in larger amounts than intact fetal nucleated cells is an alternate approach [6]. However, approaches involving such analysis face the significant challenge of differentiating fetal from maternal nucleic acids. Several strategies employing maternal nucleic acids have been proposed including: placentally expressed mRNA SNP analysis, DNA based assays that compare SNPs across chromosomes, digital PCR, and shotgun sequencing [7], [8], [9], [10], [11]. Placentally expressed mRNA present in maternal plasma has shown moderate success for detecting T21 [11]. However, because the approach is not DNA-based and requires over a thousand fold differential between placental and maternal hematopoietic cell RNA expression, the ability to expand to other chromosomal regions and assays such as Trisomy 13 and Trisomy 18, for which the current serum quad screen offers risk analysis, is very limited [11]. Previously published DNA-based strategies have demonstrated mixed performance. SNP-based approaches have had low sensitivity, and digital PCR or shotgun sequencing approaches have demonstrated high variance around measurements of fetal DNA fractions due to their fundamental reliance on an initial whole genome amplification (WGA) step, a method notorious for both under-representing and over-representing regions of a desired target with wide variance [7], [8], [9], [10]. Unfortunately, these DNA based approaches require a separate measurement of a reference chromosome or region of the genome. This causes an additional layer of experimental variance to be introduced, and makes accurate fetal DNA concentration estimates very challenging.

**Figure 1:**
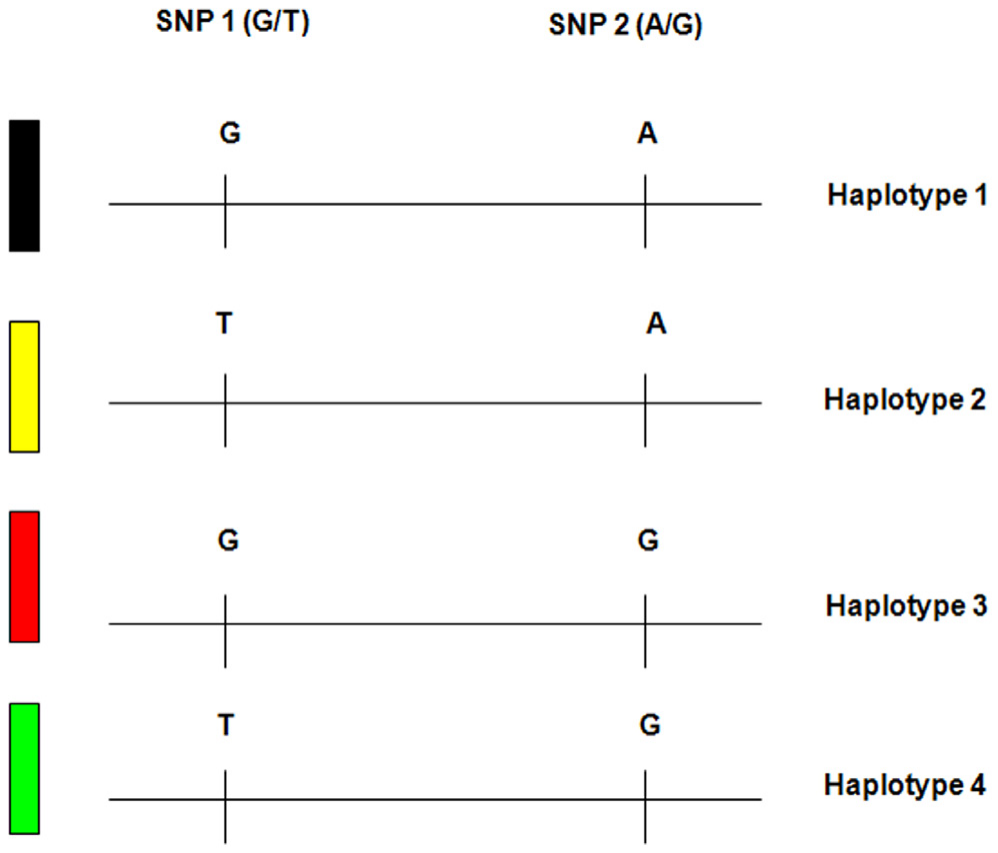
Example of two tandem SNPs (SNP1, SNP2), which constitute 4 haplotypes (Haplotype1, Haplotype2, Haplotype3, Haplotype4).

**Figure 2:**
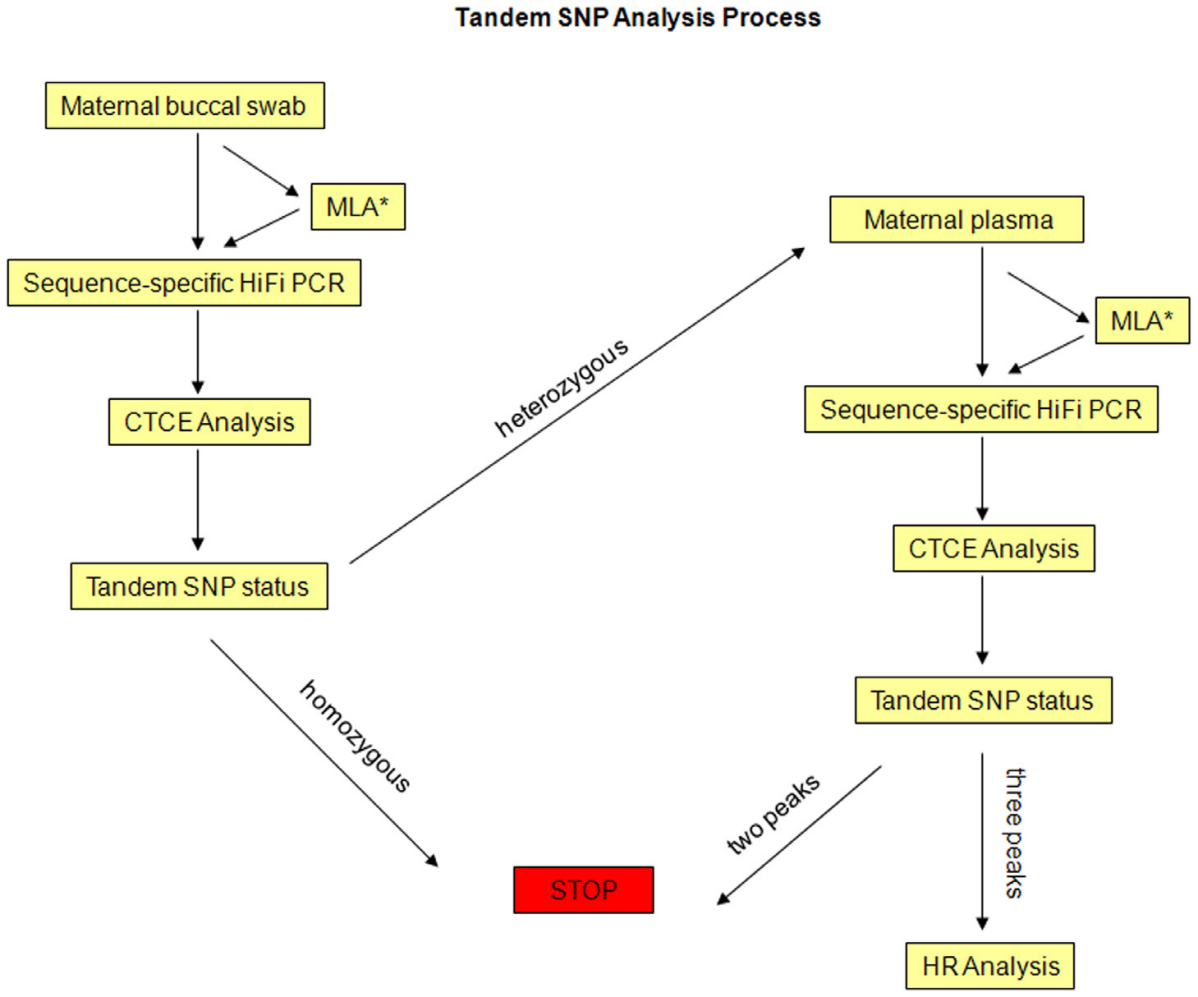
Process overview. DNA obtained from maternal buccal swab represent maternal germinal DNA. Tandem SNP sequences on chromosome 21 are amplified by MLA followed by High-Fidelity PCR (HiFi PCR) and CTCE analysis. DNA obtained from maternal plasma represents a mixture of fetal and maternal DNA. Tandem SNP sequences identified as heterozygous on maternal buccal swab are amplified on maternal plasma by MLA followed by High-Fidelity PCR (HiFi PCR) and CTCE analysis. CTCE analysis is followed by Tandem SNP evaluation to check for informativeness. Results with 3 peaks are subjected to HR analysis.

**Figure 3:**
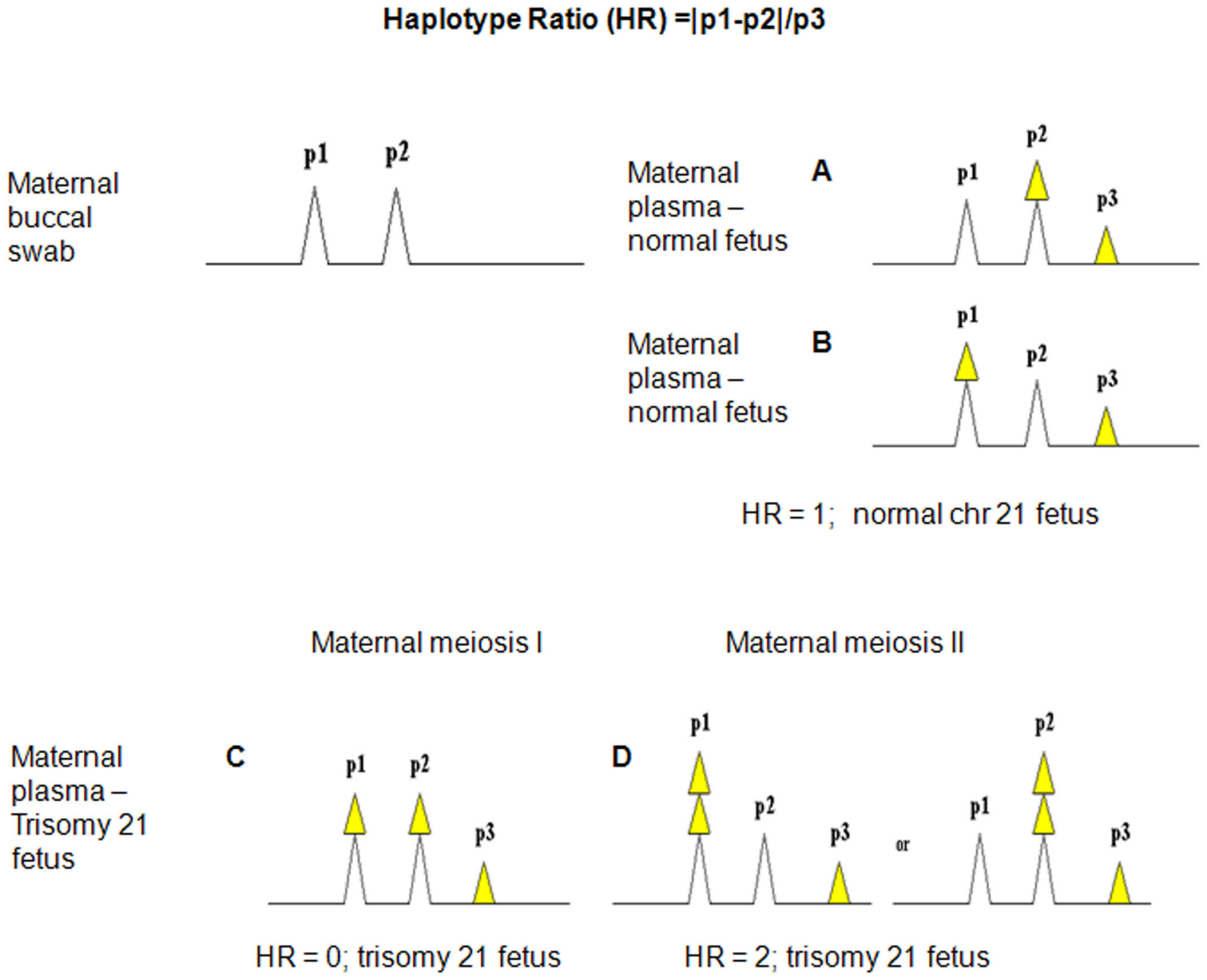
Theoretical CTCE electropherogram output from maternal buccal swab and plasma using tandem SNP analysis. Sequences exhibiting three alleles are informative. White triangles represent DNA contributed by mother. Yellow triangles represent DNA contributed by the fetus. (A) and (B) Maternal plasma sample from a mother carrying a fetus with Disomy 21 (D21) (normal) is evident by three alleles (haplotypes) all having different areas where p3, the smaller peak, is equal to the difference between p1 and p2, resulting in an HR value = 1. (C) Maternal plasma sample from a mother carrying a fetus with Trisomy 21 is evident by two equal peaks with a third smaller peak suggesting non-disjunction occurred during meiosis I, resulting in an HR value = 0, or (D) three alleles with different areas if non-disjunction occurred during meiosis II where p1 or p2 is equal to twice the area of p3, resulting in HR = 2.

Herein, we describe a novel, accurate, internally controlled, DNA-based approach to non-invasively detect fetal chromosomal abnormalities. We analyzed highly heterozygous, neighboring or “tandem” Single Nucleotide Polymorphism (SNP) sequences as short haplotypes (**Figure 1**) by CTCE, a sensitive and quantitative DNA separation technology capable of easily detecting minor DNA species present at 1% [12], [13]. The comparison of DNA obtained from maternal buccal swabs or maternal lymphocytes (representative of maternal DNA) to DNA obtained from maternal plasma (representative of a mixture of fetal and maternal DNA) (**Figure 2**) enables the determination of fetal haplotypes. Tandem SNP sequences are informative and can be used to infer fetal chromosomal dosage when the mother is heterozygous and a third paternal haplotype is detected in maternal plasma, because the maternally inherited fetal haplotype can be quantitatively compared with the paternally inherited fetal haplotype (**Figure 3**). A clear advantage of this approach is that a separate measurement of an external reference standard or reference chromosome, which would introduce additional experimental variance, is not required.

## Materials and Methods

Detailed methods are described in **Text S1**.

### Subjects and Ethics Statement

The study was approved by the Institutional Review Board (IRB) #1 for Human Research at the Medical College of Wisconsin (MCW study numbers PRO00005322 and PRO00009733). 40 high risk pregnant patients who were scheduled to undergo amniocentesis or CVS or had already had either of these procedures were recruited as subjects at Froedtert Hospital in adherence to the aforementioned IRB approved protocol. Written informed consent was obtained from each participant before blood draws and buccal swabs were obtained. Seven subjects carrying a fetus with T21 and 20 subjects carrying a fetus with normal chromosome 21 were recruited, including two subjects carrying a fetus with Trisomy 18. Karyotype analysis demonstrated that seven pregnancies carried a T21 fetus while two pregnancies carried a Trisomy18 fetus and 18 pregnancies carried a normal euploid fetus. The gestational week and maternal age varied from 9–36.1 weeks and 22–43 years respectively (**Table S1 and Table S2** online). The ethnicity of the subjects is listed also under **Table S1 and S2**. Buccal swabs from the father were also available for seven subjects. The paternal subjects were used to confirm the third peak in the maternal plasma was a paternally inherited allele.

### Multiplexed Linear Amplification

Multiplexed Linear Amplification (MLA) was performed on 18 subjects. Each sample was multiplexed with a panel of 10–15 potentially informative tandem SNP assays. Linear amplification reactions were set up using approximately 4 ng of genomic DNA, and final concentrations of 1× Buffer with 15 mM MgCl_2_ (Qiagen Inc., Valencia, California), 0.125 mM dNTPs (Fermentas Inc., Geln Burnie, Maryland), 0.04 Units/µl HotStar Taq DNA polymerase (Qiagen Inc., Valencia, California), and GC-rich primers at a final concentration of 0.6 µM, or 1× Buffer (Qiagen Inc., Valencia, California), 0.1 mM dNTPs (Fermentas Inc., Geln Burnie, Maryland), 100 ng/µl BSA (New England Biolabs, Ipswich, Massachusetts), 2 µl of Pfu Ultra II Fusion HS DNA polymerase (Qiagen Inc., Valencia, California), 0.2 µM GC-rich primers (Sigma Genosys, The Woodlands, Texas), and nuclease free water in a final reaction volume of 50 µl. The linear amplification product was used as a template in setting up the “sequence-specific” PCR as a second step. Sequence-specific PCR was set up as a high-fidelity reaction using 3 ul of linear amplification product as template (see section High-Fidelity PCR and CTCE conditions for details).

### High-Fidelity PCR and CTCE conditions

Each PCR was set up using final concentrations of 1× Pfu Ultra II Fusion HS DNA polymerase buffer (Stratagene, La Jolla, California), 0.1 mM dNTPs (Fermentas Inc., Geln Burnie, Maryland), 2 µl of the Pfu Ultra II Fusion HS DNA polymerase (Stratagene, La Jolla, California), 100 ng/µl BSA (New England Biolabs, Ipswich, Massachusetts), 0.2 µM sequence specific (Sigma Genosys, The Woodlands, Texas), genomic DNA and nuclease free water in a final reaction volume of 50 µl and run in a Mastercycler ep gradientS (Eppendorf, Westbury, New York) thermal cycler under the conditions of 95°C for 2 min followed by 42 cycles at 95°C for 30 sec, annealing at 55°C for 30 sec, extension at 72°C for 30 sec and then the final extension at 72°C for 10 min. Approximately 25 ng of genomic DNA was used for testing the maternal buccal swab. When performing direct sequence-specific PCR, approximately 3 ng of genomic DNA was used when testing maternal plasma. When linear amplification was performed prior to the sequence-specific PCR step, approximately 4 ng of genomic DNA extracted from maternal plasma was used.

All CTCE data was generated using a MegaBACE™, a high-throughput DNA analysis system (GE Healthcare Bio-Sciences Corp, Piscataway, New Jersey) [14]. Each experiment was carried out using standard MegaBACE™ buffers and MegaBACE™ LPA long-read matrix (GE Healthcare Bio-Sciences Corp, Piscataway, NJ, USA) and a 96- well low profile non-skirted plate (USA Scientific, Ocala, Florida) filled with 19 ul of deionized water and 1ul of the PCR product (each well). The theoretical melting profile of the target sequence calculated using the Winmelt simulation program was used to guide the optimal cycling temperature range (**Figure S1** online).

### CTCE data analysis and tandem SNP target sequence evaluation

CTCE .txt data files were parsed and header and electropherogram data collected for each well in the plate. The peaks from amplified DNA corresponding to the allele of interest and control amplifications were detected and integrated using standard algorithms (Sequence Analyzer v2.0 (GE Healthcare Bio-Sciences Corp, Piscataway, New Jersey)). In order to maintain quality control, parameters such as relative signal to noise ratio for the electropherogram, overall intensity of allele specific and control peaks, and inter-peak distance were calculated. Analysis was performed using the Acknowledge 3.9.1 (Biopac Systems, Inc., Goleta, California) sliding window (delta time) to define peak areas and calculate Haplotype Ratios on an Excel spreadsheet (Microsoft, Redmond, Washington).

### Statistical tests and analysis

HR 95% confidence intervals were calculated for each subject as the mean ±1.96 Standard Deviation/√3. A Receiver Operating Characteristic (ROC) curve approach was applied to the mean of the triplicate assay to ascertain what cut point would give the highest sensitivity and specificity since T21 would be called if the values were close to 0, 0.5, or 2. For the ROC curve the inverse of values less than 1 was used. However in reporting the HR results we give the range of values for which a normal and T21 result respectively would be called.

## Results

### Tandem SNP selection

A tandem SNP refers to two SNP sites within 100 bp of each other which are combined and analyzed as a single unit. To identify potentially suitable tandem SNP sequences, the International Hapmap Consortium database (NCBI build 34) was data-mined [15]. We analyzed four different files associated with the original four populations - Japanese (JPT), Han Chinese (CHB), U.S. residents with northern and western European Ancestry (CEU), and Yorubans of Ibadan, Nigeria (YRI). Potential tandem SNP sequences met the following criteria: 1) SNPs defining short haplotypes occurred within a distance of 100 base pairs from each other, 2) at least three or more haplotypes exist in all four of the above populations, and 3) haplotypes occur at high frequency (greater than 10%). One hundred and eighteen tandem SNP target sequences were designed, and reduced to 59 tandem SNP sequences by eliminating sequences which occurred in a region of known copy number variation (CNV) by comparing against the Database of Genomic Variants [16] and those which did not show an optimal separation of haplotypes according to theoretical computations of DNA melting temperatures (**Figure S1** online). Tandem SNP PCR reactions and CTCE assays were optimized for the remaining 59 tandem SNP target sequences (**Table S3** online and Materials and Methods). gDNA samples from 288 anonymous individuals (<35 years old) with no previously known genetic condition were screened. This screening verified tandem SNP haplotype frequencies in three different pools, each with 96 individuals, by high-fidelity PCR and CTCE as previously described; this analysis also demonstrated that the assay had no allelic bias during amplification [17], [18].

### Haplotype Ratio calculations to determine fetal chromosomal dosage

The maternal buccal swab or whole blood sample was first screened to identify heterozygosity at tandem SNP sequences; maternal plasma samples were processed for those that that were heterozygous. Tandem SNP sequences were considered informative when two peaks were observed in DNA extracted from maternal buccal swabs or maternal white blood cells, and three peaks were observed in DNA from maternal plasma, by CTCE analysis (**Figure 3**). Two peaks from maternal buccal swab or maternal white blood cells indicated that the mother was germline heterozygous at the tandem SNP sequence (represented by peak 1 and peak 2 in **Figure 3**). The third peak, peak 3 (**Figure 3A**, **Figure 3B**, **Figure 3C** **and** **Figure 3D**) indicated that the paternally inherited fetal haplotype was informative. When three haplotypes were observed, fetal chromosomal dosage could be inferred by calculating a HR using formula (1):
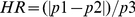
(1)where p1, p2, and p3 represent the area under peaks 1, 2, and 3 respectively in the CTCE electropherogram. HR analysis can theoretically distinguish between normal fetuses (where HR is always equal to 1) and T21 fetuses (where HR will never equal 1 but only the distinct values of 0, 0.5, or 2) as well as the stage the non-disjunction arose during maternal and paternal meiosis I and II (see **Table 1**).

**Table 1:** Theoretical haplotype ratio calculations for a normal fetus according to the etiology of the non-disjunction error during meiosis.

Chromosome 21 (fetal)	Type of meiosis	HR = (|p1−p2)|/p3)
Normal/D21	---------------------	1
T21	Maternal meiosis I	0
T21	Maternal meiosis II, Paternal meiosis I	2
T21	Paternal meiosis II	0.5

HR, Haplotype Ratio; D21, Disomy21; T21, Trisomy 21.

### Fetal Trisomy 21 detection in high risk subjects

**Figure 4:**
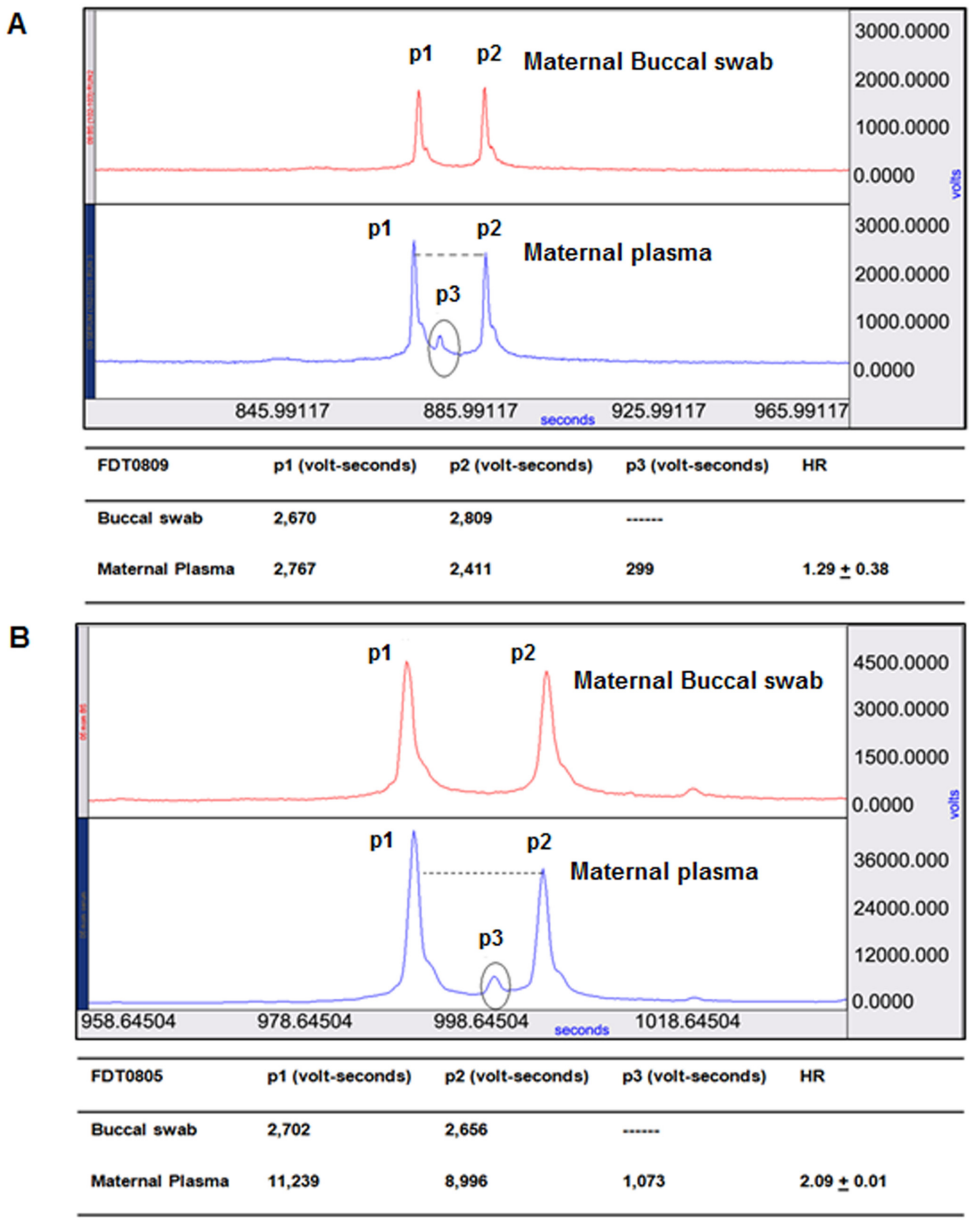
CTCE electropherogram and haplotype ratio calculations. (A) and (B) each is a combination of two panels (top panel represents buccal swab and the bottom panel represents maternal plasma) and a table that shows the haplotype ratio calculation. Dotted line in the maternal plasma sample indicates the difference in peaks p1 and p2. (A) CTCE result of FDT0809 sample. Two peaks, p1 and p2 in the top panel (derived from a buccal swab), show that the subject/mother is germline heterozygous. Third peak (p3) seen between peaks p1 and p2 in the bottom panel (maternal plasma) is identified as the third haplotype, which is paternally inherited contribution to the fetus. As the experiment was performed in triplicate, HR values were reported as the mean +/− the standard deviation in the table below the panel. Peak areas (p1, p2, p3) are reported as mean value of the triplicates. The chromosome 21 call was D21 for this subject as the HR value is approximately 1. (B) CTCE result of FDT0805 sample. Two peaks, p1 and p2 in the top panel (buccal swab) show that the subject/mother is germline heterozygous. The chromosome 21 call is T21 for this subject as the HR value is approximately 2. Chromosome 21 calls by HR value were confirmed by fetal karyotype results.

Forty high risk pregnant women were enrolled as subjects at Froedtert Hospital in strict adherence to MCW IRB protocols. One sample was lost during extraction (FDT0823), six samples did not have enough DNA to proceed with analysis due to low recovery (<2 ng), these were (FDT0814, FDT0815, FDT0828, FDT0829, FDT0831, FDT0838). Six samples were not informative at the 9–16 tandem SNP assays that were tested for informativeness before running out of sample (FDT0811, FDT0819, FDT0820, FDT0825, FDT0826, FDT0830) (see **Text S1** online). A total of 27 subjects were assessable, 18 subjects were pregnant women carrying a normal fetus, two were pregnant women carrying a Trisomy 18 (T18) fetus and seven were pregnant women carrying a T21 fetus. The two subjects carrying a T18 fetus served as additional controls because all of the tandem SNP sequences used in this study were targeted to chromosome 21. Haplotype ratios were determined and chromosome 21 calls were made for the 27 patient plasma samples based on the HR values as either T21 or Disomy 21 (D21). The HR values for 27 study samples are listed under **Table S4** online. The 95% CI for T21 showed no overlap with the D21 and vice versa. Further, 100% sensitivity and 100% specificity was obtained for the mean triplicate values when HR values lying between 3/5 and 5/3 were called as D21 and any values outside this range were called as T21. Examples of HR analysis resulting in the detection of a normal or D21 fetus and a T21 fetus are shown in **Figure 4A** and **Figure 4B**. The electropherogram outputs for remaining subjects are shown in **Figure S2** online. Ten tandem SNP sequences were informative on 27 study subjects (see **Table S5** online). All 27 samples were correctly called and confirmed by either amniocentesis, CVS, or by postnatal karyotype.

Following HR analysis and unblinding of the study, the tandem SNP regions were analyzed again with software developed to automate the selection process by looking at phased theoretical haplotype frequencies (data not shown) using NCBI build 36 of HapMap data (Phase II+III). Based upon this analysis, one tandem SNP target (rs432114–rs365433) which showed a third (fetal) peak on subject FDT0604 was not predicted to be highly informative. It was later determined that a third SNP resides between the two original SNPs of interest although it is unknown if this was the third, informative haplotype. Detection of novel variants is a previously recognized limitation of CTCE when being utilized as a genotyping method [19]. A second tandem SNP target (rs8134080–rs2831524) which showed a third peak was therefore analyzed for subject FDT0604 and the HR value is reported in **Table S4** online.

### Multiplexed Linear Amplification (MLA)

A major challenge of fetal DNA analysis using maternal plasma is the limited amounts of fetal DNA available per maternal blood sample (i.e. typically 10–20 ml of blood). This in turn limits the number of possible assays that can be analyzed. Various methods for global amplification of DNA target molecules via whole genome amplification (WGA) are frequently utilized [20], [21], [22], [23], [24]. However, a recognized and critical limitation of WGA for this kind of analysis is the frequent introduction of non-specific amplification artifacts, incomplete coverage of loci, and the propensity to generate products that are preferentially amplified, occasionally resulting in biased allelic representation of genomic sequences in the product.

To amplify our tandem SNP target sequences, we optimized a multiplexed, linear amplification step. 5′ flap sequences (e.g., a 54-mer oligonucleotide rich in G and C bases) attached to the 5′ end of a primer is highly efficient during linear PCR (by using a single primer to amplify DNA template in one direction, because there is no partner primer, the amplification is linear rather than exponential). Indeed, frequently yields greater than 100% are observed, suggesting multiple primer initiations per parent DNA sequence. We hypothesized that primers with a GC-rich tail could be multiplexed and linearly amplified to avoid primer interactions without introducing allelic bias during amplification (see Materials and Methods for Multiplexed Linear Amplification). Reconstruction experiments comparing MLA to direct PCR are further described in **Text S1** (see **Figure S3** and **Figure S4** online).

**Figure 5:**
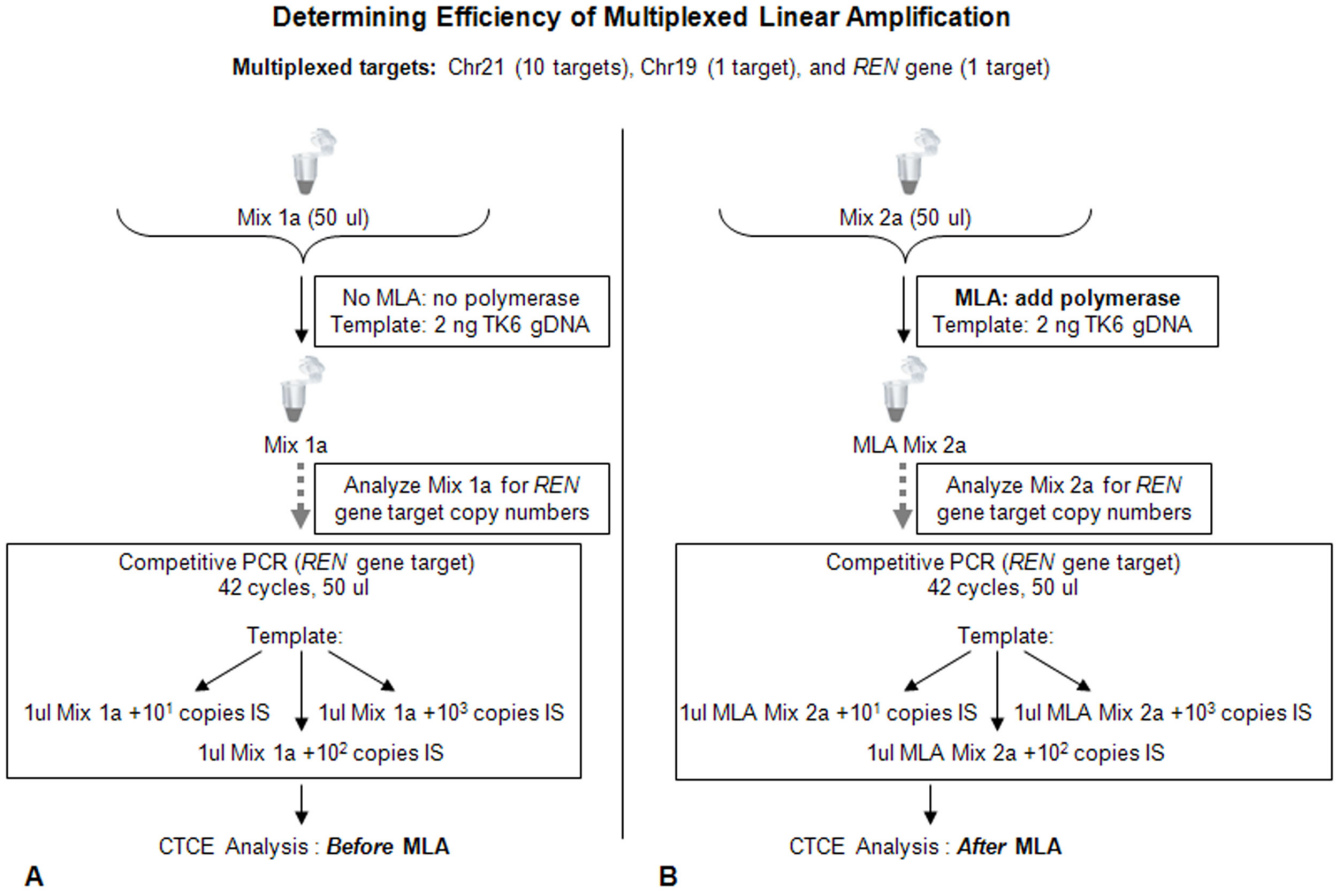
Calculating yield for Multiplexed Linear Amplification (MLA). A multiplexed linear PCR was setup with 12 primer sequences targeting 12 different targets including ten tandem SNP target sequences from chromosome 21, one target from chromosome 19, and one target on exon 1 of the *REN* gene. 2 ng genomic DNA from TK6 cells was used as template. (A) Mix 1a included template, buffer, and all primers but no polymerase was added and did not undergo linear amplification and denoted as a “before MLA” mix. (B) Mix 2a was identical to Mix 1a except for the addition of polymerase and 45 cycles of linear amplification and is denoted as an “after MLA” mix. One ul of each mix was then quantified for copy numbers of the *REN* gene target sequence by competitive PCR using an artificial mutant sequence spiked in at three different concentrations (10^1^, 10^2^, and 10^3^ copies) followed by CTCE analysis. All “before MLA” and “after MLA” mixes were set up in triplicate (mix 1a, 1b, and 1c and did not include polymerase and did not undergo cycling, similarly, mix 2a, 2b, and 2c did include polymerase and did undergo cycling). All competitive PCR reactions were performed in triplicate for all six mixes. Comparison of *REN* gene target copy numbers before and after linear amplification divided by the number of cycles led to estimation of an average yield per cycle of linear amplification (**Table S6** online).

The yield (efficiency) of linear amplification was determined by assessing the copy number of the target sequences prior to and following linear amplification. Copies of the human renin gene (*REN* exon 1) were quantified by competitive PCR where a known amount of an artificial mutant PCR product containing a single base pair difference from the wild-type was added in to a *REN* gene sequence-specific master mix along with 1 ul of the sample “before linear amplification” or 1 ul of the sample “after linear amplification” to serve as an internal standard (see **Text S1** online) [25]. The internal standard was spiked in at 10 copies, 100 copies and 1,000 copies per reaction to enable quantification of DNA copies before and after linear amplification. After 45 cycles, competitive PCR products were analyzed by CTCE. Yields of greater than 100% per cycle at the renin gene locus was observed when multiplexed with 12 GC-rich primers (see **Figure 5A**, **Figure 5B**) and (**Table S6** online).

**Figure 6:**
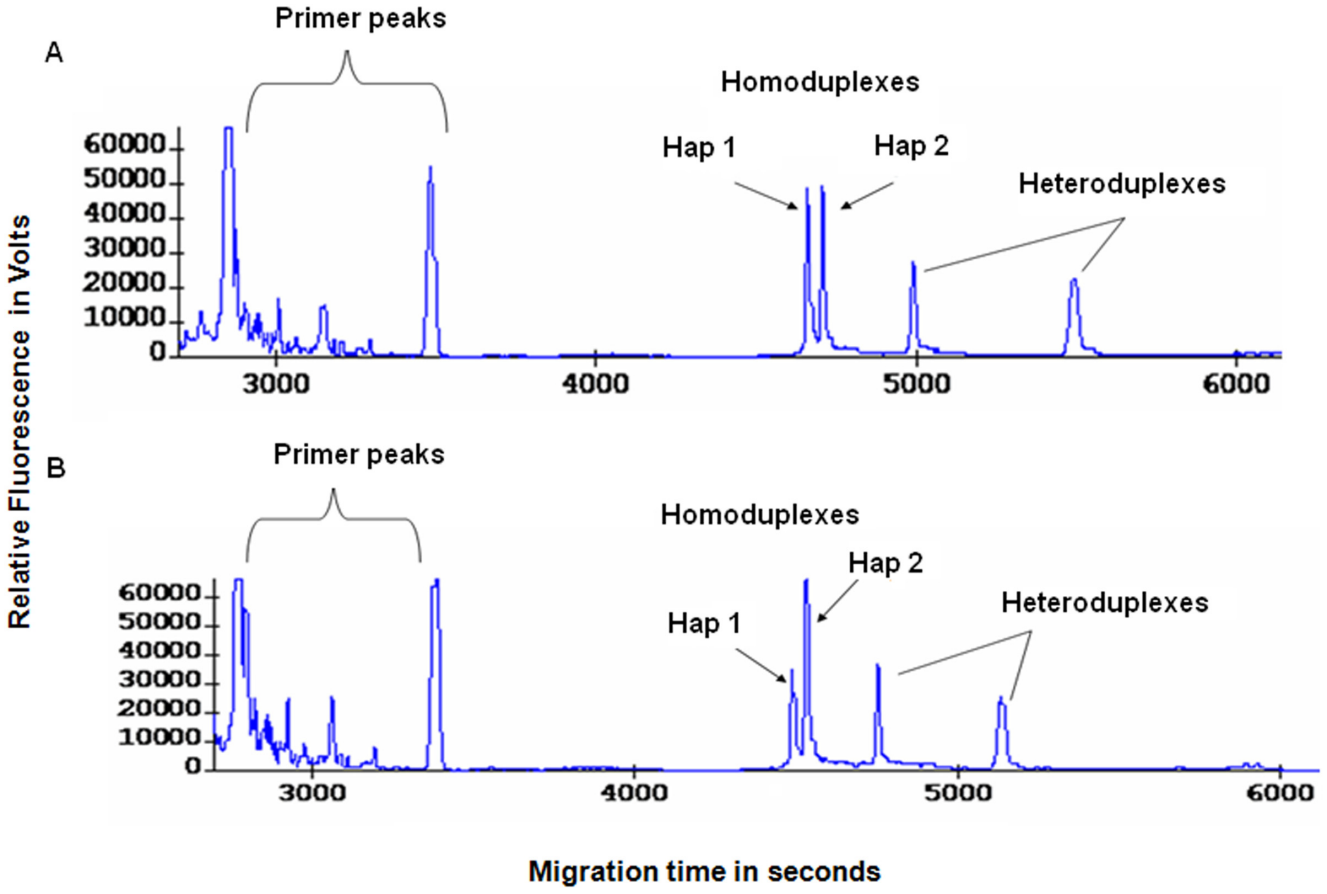
Allelic bias is present following ligation-mediated PCR (LM-PCR) but not Multiplexed Linear Amplification (MLA). (A) Starting from 6.25 ng genomic DNA, electropherogram of a heterozygous sequence at tandem SNP location rs11088086–rs2251447 following LM-PCR. Allele 2 was clearly preferentially amplified. (B) Starting from 6.25 ng genomic DNA, electropherogram of a heterozygous sequence at tandem SNP location rs11088086–rs2251447 following MLA. No allelic bias was observed for MLA with six assays.

We then compared MLA products with samples that had been amplified by ligation-mediated PCR (LM-PCR), a commonly used technique in WGA [24] (see Materials and Methods). Significant allelic differences were observed when LM-PCR products were used as template for sequence-specific HiFi PCR of heterozygous tandem SNP sequences and CTCE analysis in all tandem SNP sequences (**Figure 6A**). Allelic bias was not observed when MLA products were used as template (**Figure 6B**).

## Discussion

This study supports the potential for tandem SNP analysis to be used as an effective and accurate means for non-invasive diagnosis of T21. Tandem SNP sequences are short (less than or equal to 100 bp size), which is preferred in a targeted sequence-specific approach because fetal DNA fragments in maternal circulation are short. These short haplotypes are informative when a third haplotype is present in maternal plasma. The algorithm for analysis is straightforward. A HR is determined, which approximately compares the maternally inherited contribution to the fetus, |p1−p2|, to the paternally inherited contribution to the fetus, p3.

The tandem SNP approach has several distinct advantages over other approaches which also analyze circulating fetal nucleic acids in maternal blood. First, HR analysis tests for discrete outputs; values of either 1 (normal), 0 (trisomy), 0.5 (trisomy or deletion), or 2 (trisomy) are the only expected HR output. The *a priori* hypothesis of discrete HR outputs permits the establishment of population-based confidence intervals (standards) around these values, a significant advantage over approaches that test if fetal DNA concentrations are different between a chromosome of interest and a reference chromosome or genomic region. Because fetal DNA concentrations are highly variable and influenced by many factors (including gestational age, fetal size, etc.), approaches which test for differences between fetal DNA concentrations at a chromosome of interest (i.e. chromosome 21) and a reference chromosome/genomic region must be performed at an individual level for each patient, and is statistically more challenging. In this study, the ROC curve would have allowed a cut point anywhere between 1.67 and 1.96 without changing sensitivity or specificity but additional studies will be required to establish the true threshold.

Second, because an internal comparator exists for every informative tandem SNP sequence, the experimental variability introduced through the tandem SNP approach is smaller than other approaches requiring an external reference. This can be seen by first looking at the difference on the HR numerator (the maternally inherited fetal haplotype, |p1−p2|). The difference of the two maternal haplotypes is the sum of the variances minus twice the covariance (positive since SNPs within this chromosome are positively correlated) between the two haplotypes. This is quite different when an independent external comparator is used. For example, a previous study employed a single SNP approach to calculate fetal DNA fractions on chromosome 21 [7]. In this study, the assay was informative when the mother was homozygous for a SNP and the fetus was heterozygous, contributing a second allele. The fetal DNA concentrations calculated on chromosome 21 were then compared with fetal DNA concentrations on a reference chromosome (chromosome 13) [7]. Assuming the variability of the SNPs on the different chromosomes are the same, one would have the sum of the variances that would exceed the variance observed in our approach. Furthermore, the HR denominator (p3) serves as an internal standard or reference and as such will be correlated with the numerator. The variance of a ratio R/S using a Taylor series expansion is approximately
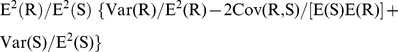
(2)where E (.) denotes the expected value, Var (.) the variance and Cov (.,.) the covariance. Since the covariance will be positive again this ratio will be less than if an independent external reference was used. To summarize, our internal standard reference (p3) allows for a reduction in variability from two sources: the variability of the numerator (R) and the overall variance because the covariance of the measurements lessens the variability. This initial study in pregnant women supports the notion that having an internal comparator can lead to accurate measurements of fetal DNA concentrations; 7 of 7 T21 fetuses were correctly identified and 20 of 20 D21 subjects were correctly identified.

Third, because the tandem SNP approach is targeted to potentially informative tandem SNP sequences, a limited number of regions are required for analysis. This simplifies the process, limits costs, and enables extension to large batch processing with quick turnaround. Methods employing a whole genome shotgun approach are costly both in dollars and time (although the data can be obtained relatively quickly, processing the data is quite time consuming at present). It should be noted that previous publications suggest minor alleles present at 1% are easily and reproducibly detected by CTCE [12], [13]. Our results are consistent with these previous studies and all of the informative results described in this study are based off of paternal allele frequencies greater than 1%. Because the third allele (paternally inherited allele) would serve as the minor allele, this would translate to a fetal DNA fraction limit of detection of 2% for a euploid fetus and 3% for a T21 fetus.

Fourth, unlike Short Tandem Repeats (STRs), which would also be potentially highly informative, tandem SNP sequences can be amplified with high fidelity and can also be multiplexed to a high degree.

Finally, the tandem SNP approach has wide applicability for additional chromosomal defects, i.e., it could be used for the prenatal detection of trisomy 18, trisomy 13, and it could be extended to other regions on the genome and chromosomal abnormalities. Furthermore, the tandem SNP approach is not platform dependent. The general strategy of targeted analysis of tandem SNP sequences can be performed on multiple platforms, including technologies with high throughput such as next-generation sequencing.

## Supporting Information

Figure S1 DNA melting map of CTCE target sequence covering tandem SNPs (rs961301–rs2830208). Winmelt software (BioRad, Hercules, California) was used to identify the melting profile of the DNA sequences which calculates the thermal stability and denaturation behaviour of double-stranded DNAs and RNAs up to a length of 1,000 base pairs. The Winmelt algorithm is based on recursive generation of conditional and a priori probabilities for base stacking. The melting profile output of the program can be compared directly to the experimental results and thus this program is used to optimize the experimental conditions prior to CTCE analysis. All three haplotypes can be theoretically separated according to DNA melting temperature. Black line indicates haplotype 1 (C, C). Yellow line indicates haplotype 2 (C,T). Red line indicates haplotype 3 (T, C). This graph shows that the melting temperature for this target sequence is approximately 66°C.
(0.21 MB TIF)

Figure S2 Electropherogram for 25 study samples. Screenshots of Acknowledge software output for electropherograms of 25 maternal plasma samples (buccal swab and plasma of each subject). Each subject's result/plot is a combination of two panels where the top panel represents the buccal swab and the bottom panel represents maternal plasma. Along the x-axis is migration time (seconds/minutes) and the y-axis is Relative Fluorescence (volts). Peak inside the circle in bottom panel is the fetal peak (paternal contribution). Peak areas for maternal plasma are measured to calculate HR values (Table S4 online).
(3.89 MB TIF)

Figure S3 Reconstruction Experiments. Tandem SNPs rs2832141–rs2246777 were analyzed by CTCE in an infant-maternal pair. The top panel (A) is the electropherogram of only maternal DNA isolated from white blood cells (WBC) followed by (B) the electropherogram of DNA isolated from cord blood of a confirmed T21 infant, and (C) and (D) are electropherograms depicting the infant-maternal mixtures at 10% and 5%, respectively.
(1.54 MB TIF)

Figure S4 Box Plot for comparing Direct PCR to MLA in Reconstruction Experiments. The HRs are approximately 1 for the euploid reconstruction mixtures and close to 0 for the T21 reconstruction mixtures, irrespective of the protocol (Direct PCR or MLA) used. This demonstrates that HRs calculated following MLA are not significantly different from HRs obtained following Direct PCR. The simulated concentrations were aimed at 2.5%, 5%, 10%, 20%, and 40% for both a confirmed T21 infant - maternal mixture and a euploid infant - maternal mixture. True paternal percentages were approximately in the range of 2.4–4% for 2.5% mixture, 3.7–7% for the 5% mixture, 3–9% for the 10% mixture, 6–14% for the 20% mixture and 17–24% for the 40% mixture as estimated by CTCE.
(0.13 MB TIF)

Table S1 Maternal subjects assessed for Haplotype Ratios.
(0.05 MB DOC)

Table S2 Non-informative subjects.
(0.04 MB DOC)

Table S3 Primer sequences for 58 tandem SNP pairs.
(0.08 MB DOC)

Table S4 Maternal plasma DNA analysis using Haplotype Ratio calculations.
(0.06 MB DOC)

Table S5 Informative tandem SNP assays and subject number.
(0.03 MB DOC)

Table S6 MLA followed by CTCE analysis. Starting from approximately 9 copies per tube (Mix 1a, 1b, 1c) following MLA, approximately 47 copies were created per cycle or a yield greater than 500%.
(0.06 MB DOC)

Text S1 Supporting Information.
(0.07 MB DOC)
