# Supplementary figures and images for: Non-Invasive Prenatal Detection of Trisomy 21 Using Tandem Single Nucleotide Polymorphisms
Source: PLoS One. 2010 Oct 8;5(10):e13184. doi: 10.1371/journal.pone.0013184 (PMC2951898; doi:10.1371/journal.pone.0013184)

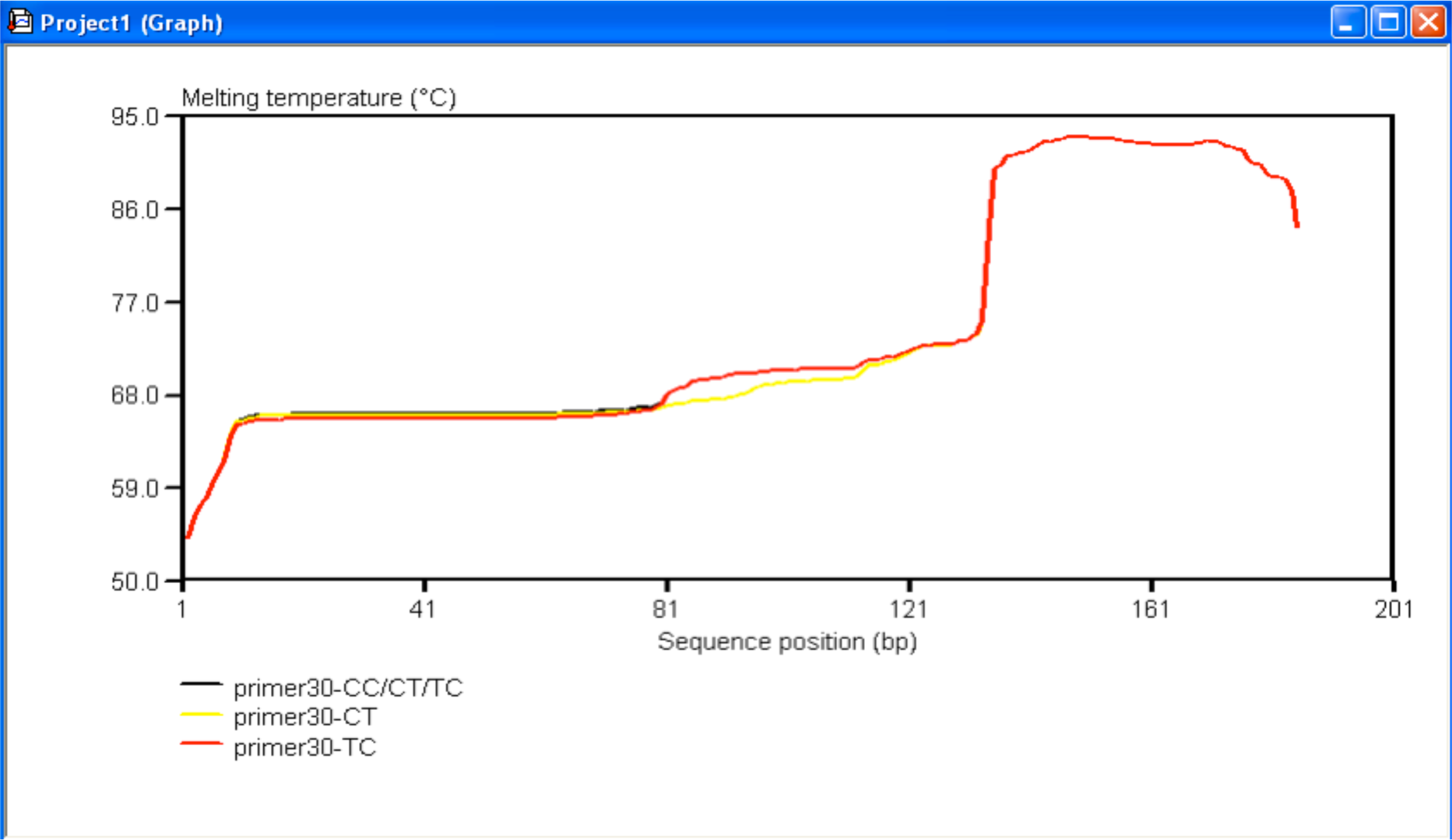

Supplement: Figure S1 — DNA melting map of CTCE target sequence covering tandem SNPs (rs961301–rs2830208). Winmelt software (BioRad, Hercules, California) was used to identify the melting profile of the DNA sequences which calculates the thermal stability and denaturation behaviour of double-stranded DNAs and RNAs up to a length of 1,000 base pairs. The Winmelt algorithm is based on recursive generation of conditional and a priori probabilities for base stacking. The melting profile output of the program can be compared directly to the experimental results and thus this program is used to optimize the experimental conditions prior to CTCE analysis. All three haplotypes can be theoretically separated according to DNA melting temperature. Black line indicates haplotype 1 (C, C). Yellow line indicates haplotype 2 (C,T). Red line indicates haplotype 3 (T, C). This graph shows that the melting temperature for this target sequence is approximately 66°C. (0.21 MB TIF) [file pone.0013184.s001.tif]

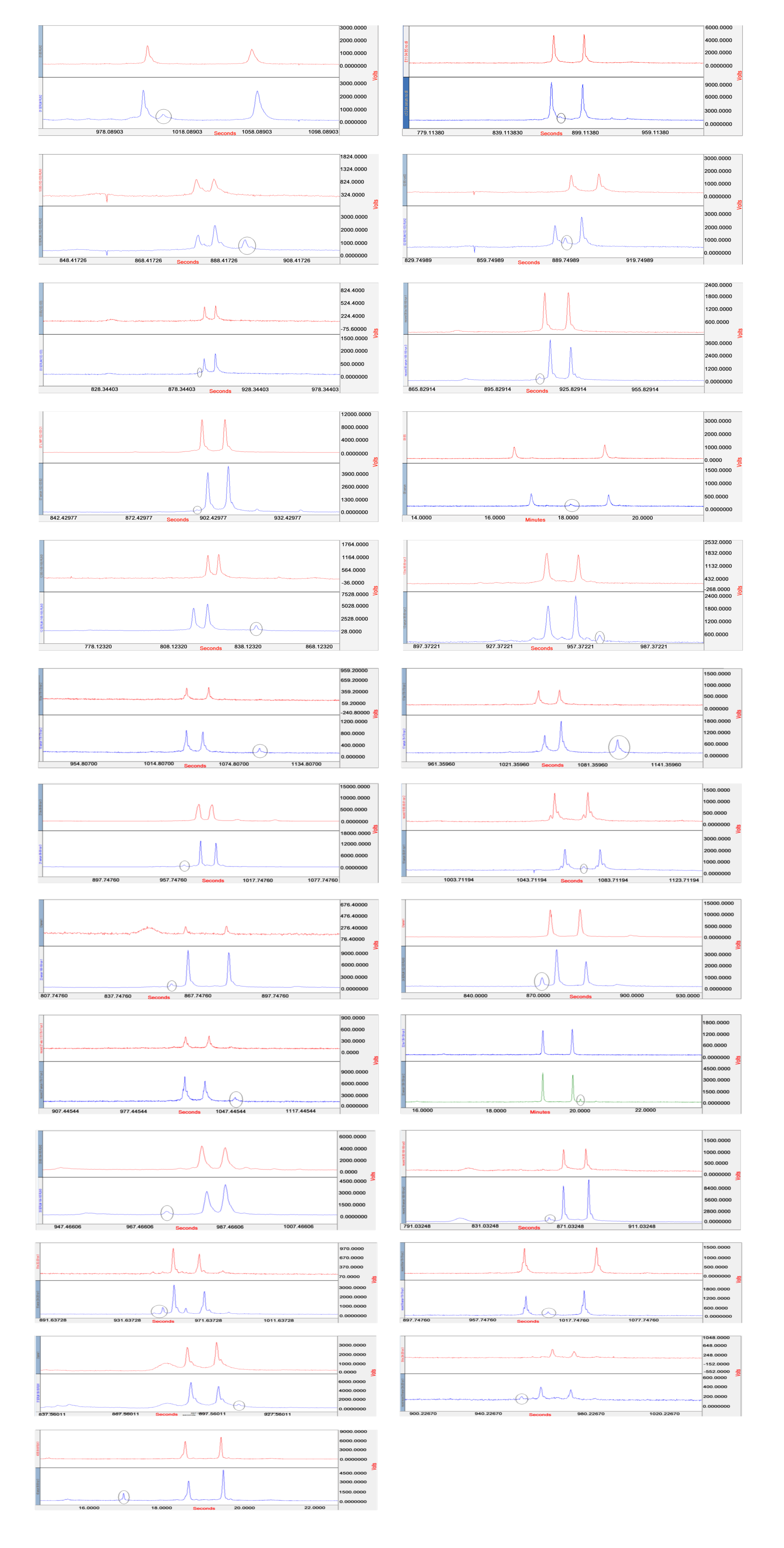

Supplement: Figure S2 — Electropherogram for 25 study samples. Screenshots of Acknowledge software output for electropherograms of 25 maternal plasma samples (buccal swab and plasma of each subject). Each subject's result/plot is a combination of two panels where the top panel represents the buccal swab and the bottom panel represents maternal plasma. Along the x-axis is migration time (seconds/minutes) and the y-axis is Relative Fluorescence (volts). Peak inside the circle in bottom panel is the fetal peak (paternal contribution). Peak areas for maternal plasma are measured to calculate HR values (Table S4 online). (3.89 MB TIF) [file pone.0013184.s002.tif]

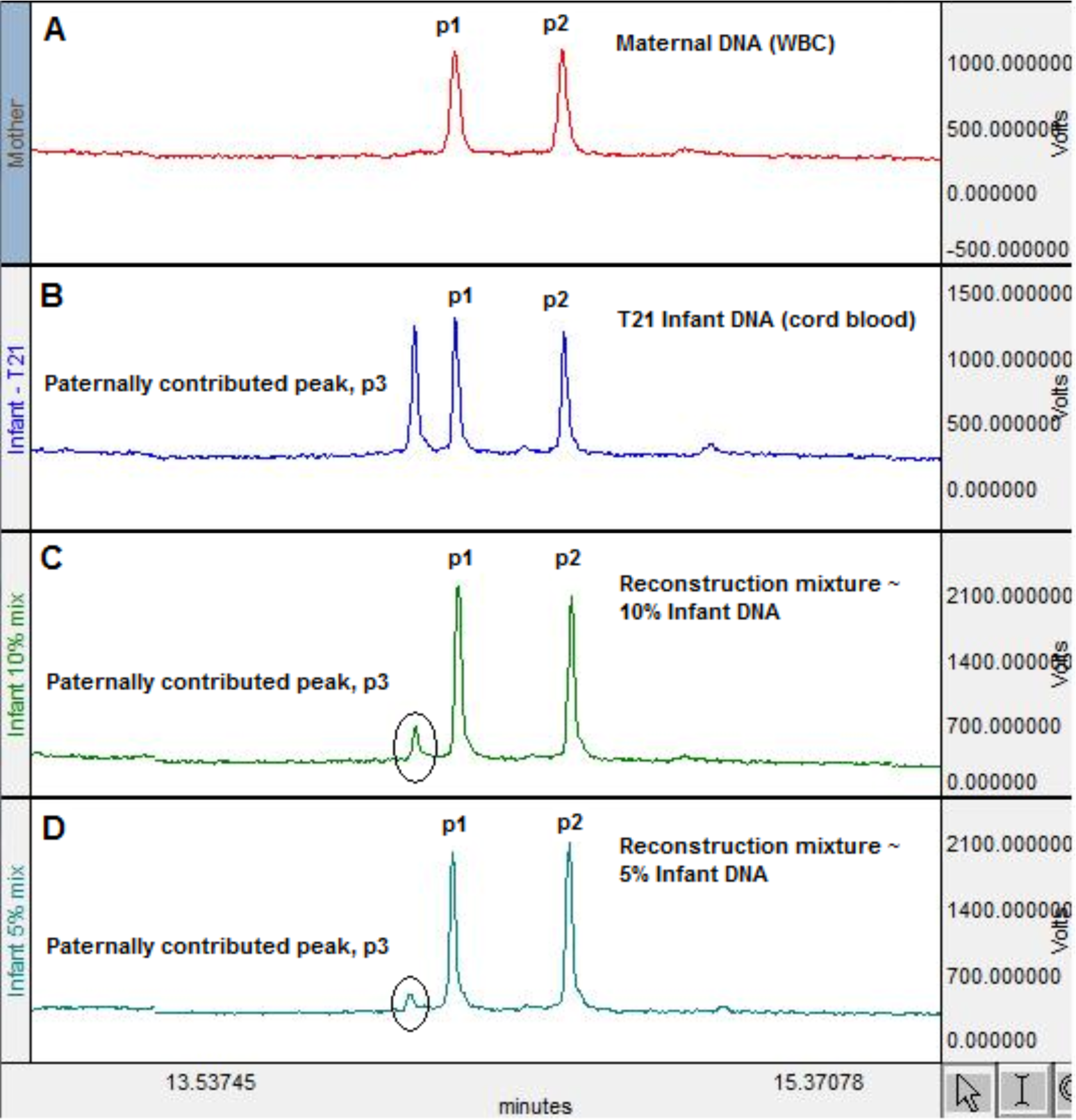

Supplement: Figure S3 — Reconstruction Experiments. Tandem SNPs rs2832141–rs2246777 were analyzed by CTCE in an infant-maternal pair. The top panel (A) is the electropherogram of only maternal DNA isolated from white blood cells (WBC) followed by (B) the electropherogram of DNA isolated from cord blood of a confirmed T21 infant, and (C) and (D) are electropherograms depicting the infant-maternal mixtures at 10% and 5%, respectively. (1.54 MB TIF) [file pone.0013184.s003.tif]

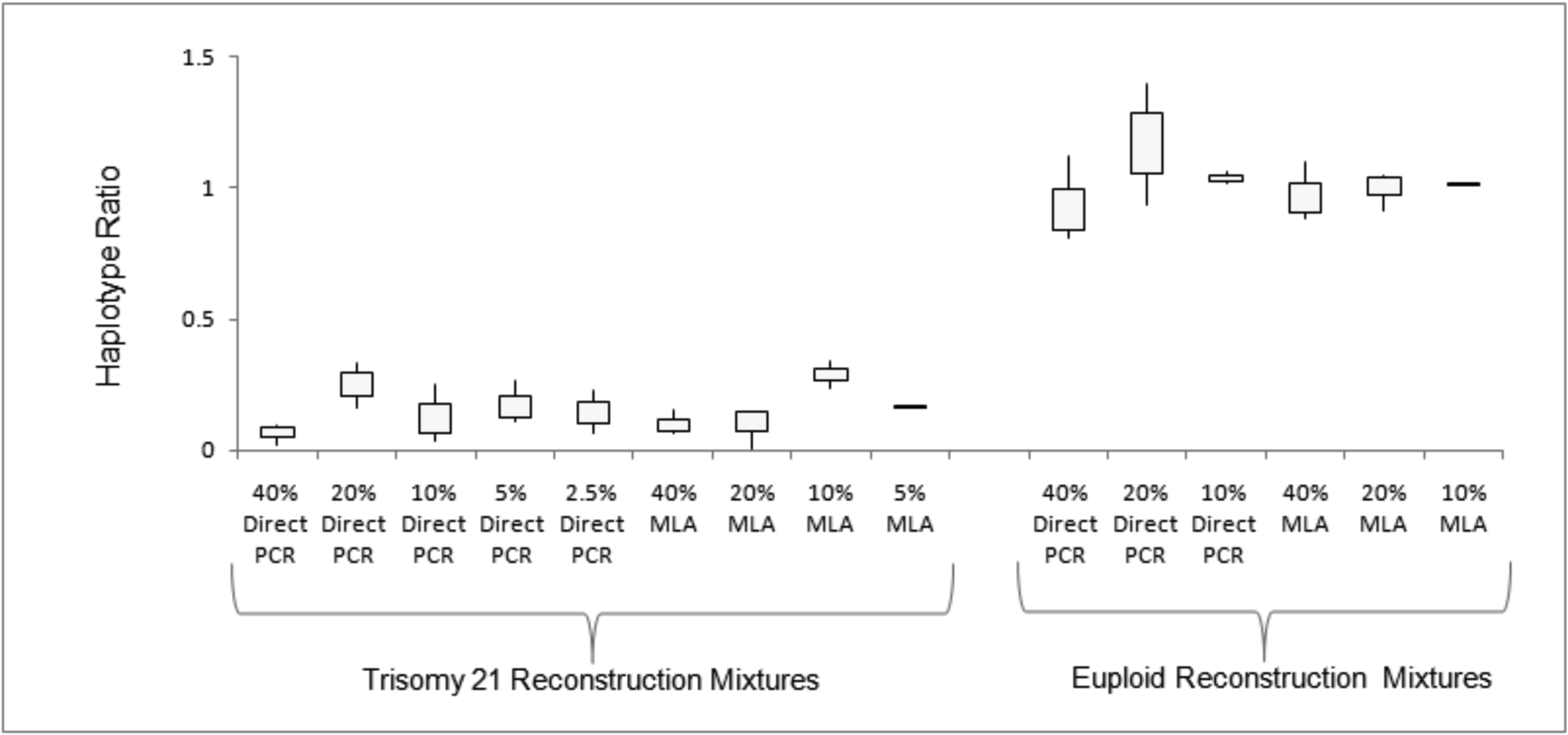

Supplement: Figure S4 — Box Plot for comparing Direct PCR to MLA in Reconstruction Experiments. The HRs are approximately 1 for the euploid reconstruction mixtures and close to 0 for the T21 reconstruction mixtures, irrespective of the protocol (Direct PCR or MLA) used. This demonstrates that HRs calculated following MLA are not significantly different from HRs obtained following Direct PCR. The simulated concentrations were aimed at 2.5%, 5%, 10%, 20%, and 40% for both a confirmed T21 infant - maternal mixture and a euploid infant - maternal mixture. True paternal percentages were approximately in the range of 2.4–4% for 2.5% mixture, 3.7–7% for the 5% mixture, 3–9% for the 10% mixture, 6–14% for the 20% mixture and 17–24% for the 40% mixture as estimated by CTCE. (0.13 MB TIF) [file pone.0013184.s004.tif]
