# Supplementary material for: Non-Invasive Prenatal Detection of Trisomy 21 Using Tandem Single Nucleotide Polymorphisms
Source: PLoS One. 2010 Oct 8;5(10):e13184. doi: 10.1371/journal.pone.0013184 (PMC2951898; doi:10.1371/journal.pone.0013184)
Supplement: Table S1 — Maternal subjects assessed for Haplotype Ratios. (0.05 MB DOC) [file pone.0013184.s005.doc]

Table S1. Maternal subjects assessed for Haplotype Ratios.

| **Subject #** | **Volume of blood drawn (ml)** | **Maternal Age (years)** | **Race** | **Ethnicity** | **Gestational Week (weeks)** |
| --- | --- | --- | --- | --- | --- |
|
|
| FDT0601 | 8 | 35 | Asian | Non-hispanic/Non-Latino | 9 |
| FDT0602 | 6 | 30 | White | Non-hispanic/Non-Latino | 14.3 |
| FDT0603 | 8 | 26 | White | Non-hispanic/Non-Latino | 35 |
| FDT0604 | 14 | 29 | White | Non-hispanic/Non-Latino | 27.6 |
| FDT0705 | 4 | 42 | White | Non-hispanic/Non-Latino | 25 |
| FDT0706 | 12 | 40 | Asian | Other | 29.5 |
| FDT0807 | 8 | 33 | White | Non-hispanic/Non-Latino | 31.3 |
| FDT0808 | 12 | 40 | White | Non-hispanic/Non-Latino | 35.5 |
| FDT0809 | 8 | 43 | White | Non-hispanic/Non-Latino | 12.7 |
| FDT0810 | 8 | 40 | Unknown | Unknown | 15 |
| FDT0812 | 12 | 38 | White | Non-hispanic/Non-Latino | 12.6 |
| FDT0813 | 12 | 40 | White | Non-hispanic/Non-Latino | 14.5 |
| FDT0816 | 12 | 25 | White | Other | 26 |
| FDT0817 | 12 | 30 | White | Other | 13.5 |
| FDT0818 | 12 | 39 | White | Non-hispanic/Non-Latino | 11.4 |
| FDT0821 | 12 | 28 | White | Non-hispanic/Non-Latino | 11.4 |
| FDT0822 | 12 | 33 | White | Other | 33.5 |
| FDT0824 | 16 | 41 | Other | Non-hispanic/Non-Latino | 15.4 |
| FDT0827 | 12 | 32 | Unknown | Unknown | 30.2 |
| FDT0832 | 20 | 37 | White | Non-hispanic/Non-Latino | 14.6 |
| FDT0833 | 20 | 24 | White | Non-hispanic/Non-Latino | 36.1 |
| FDT0834 | 20 | 33 | White | Non-hispanic/Non-Latino | 13.4 |
| FDT0835 | 20 | 22 | White | Non-hispanic/Non-Latino | 20.3 |
| FDT0836 | 20 | 32 | White | Non-hispanic/Non-Latino | 11.5 |
| FDT0837 | 20 | 33 | White | Non-hispanic/Non-Latino | 13.3 |
| FDT0839 | 20 | 29 | No info | No info | 19.6 |
| FDT0840 | 20 | 32 | No info | No info | 17 |
|  | | | | | |
