# Supplementary material for: Non-Invasive Prenatal Detection of Trisomy 21 Using Tandem Single Nucleotide Polymorphisms
Source: PLoS One. 2010 Oct 8;5(10):e13184. doi: 10.1371/journal.pone.0013184 (PMC2951898; doi:10.1371/journal.pone.0013184)
Supplement: Table S2 — Non-informative subjects. (0.04 MB DOC) [file pone.0013184.s006.doc]

Table S2. Non-informative subjects.

| **Subject #** | **Volume of blood drawn (ml)** | **Maternal Age (years)** | **Race** | **Ethnicity** | **Gestational Week (weeks)** | **# of assays tested on maternal plasma** | **Tandem SNP assay #s ^1^** |
| --- | --- | --- | --- | --- | --- | --- | --- |
| FDT0811 | 12 | 35 | White | Non-Hispanic / Non-Latino | 15.2 | 16 | rs1735976-rs2827016, rs447349-rs2824097, rs418989- rs13047336, rs4817013-rs7277036, rs2831244-rs9789838, rs8132769-rs2831440, rs8134080-rs2831524, rs11088086-rs2251447, rs2832141-rs2246777, rs2776266-rs2835001, rs7281674-rs2835316, rs2835735-rs2835736, rs2836550-rs2212596, rs418359-rs2836926, rs2837296-rs2837297, rs2837381-rs4816672 |
| FDT0819 | 12 | 26 | White | Non-Hispanic / Non-Latino | 12.6 | 14 | rs2822731-rs2822732, rs1735976-rs2827016, rs11909758-rs9980111, rs2831244-rs9789838, rs8134080-rs2831524, rs11088086-rs2251447, rs2832040-rs11088088, rs2832141-rs2246777, rs2834485-rs3453, rs13047304-rs13047322, rs9980072-rs8130031, rs418359-rs2836926, rs385787-rs367001, rs2837381-rs4816672 |
| FDT0820 | 12 | 40 | White | Non-Hispanic / Non-Latino | 11.3 | 16 | rs2822654-rs1882882, rs961301-rs2830208, rs2174536-rs458076, rs4817219-rs4817220, rs2250911-rs2250997, rs2832040-rs11088088, rs2832141-rs2246777, rs933121-rs933122, rs9974986-rs2834703, rs2776266-rs2835001, rs7281674-rs2835316, rs13047304-rs13047322, rs2835735-rs2835736, rs385787-rs367001, rs367001-rs386095, rs2837296-rs2837297 |
| FDT0825 | 12 | 33 | Asian (Indian) | Non-Hispanic / Non-Latino | 14.5 | 10 | rs447349-rs2824097, rs4143392- rs4143391, rs7509629-rs2828358, rs4817219-rs4817220 , rs2833734-rs2833735, rs2834485-rs3453, rs7281674-rs2835316, rs2836550-rs2212596, rs9980072-rs8130031, rs2837381-rs4816672 |
| FDT0826 | 12 | 29 | White | Non-Hispanic / Non-Latino | 17.2 | 13 | rs2822731-rs2822732, rs11909758-rs9980111, rs961301-rs2830208, rs2174536-rs458076, rs2832040-rs11088088, rs2833734-rs2833735, rs2834485-rs3453, rs9974986-rs2834703, rs7281674-rs2835316, rs13047304-rs13047322, rs2835735-rs2835736, rs418359-rs2836926, rs385787-rs367001 |
| FDT0830 | 16 | 35 | White | Non-Hispanic / Non-Latino | 10.5 | 9 | rs1735976-rs2827016, rs418989- rs13047336, rs4817013-rs7277036 , rs2174536-rs458076, rs2250911-rs2250997 , rs11088086-rs2251447, rs13047304-rs13047322, rs418359-rs2836926 |
| **^1^**dbSNP accession numbers of two tandem SNPs (separated by dash) and different tandem SNP pair #s (separated by a comma). | | | | | | | |
