# Supplementary material for: Non-Invasive Prenatal Detection of Trisomy 21 Using Tandem Single Nucleotide Polymorphisms
Source: PLoS One. 2010 Oct 8;5(10):e13184. doi: 10.1371/journal.pone.0013184 (PMC2951898; doi:10.1371/journal.pone.0013184)
Supplement: Table S3 — Primer sequences for 58 tandem SNP pairs. (0.08 MB DOC) [file pone.0013184.s007.doc]

Table S3. Primer sequences for 58 tandem SNP pairs.

| **Tandem SNP #^1^** | **Forward primer (5' -3')** | **Reverse primer (5' -3')** |
| --- | --- | --- |
| rs7277033-rs2110153 | TCCTGGAAAACAAAAGTATT | AACCTTACAACAAAGCTAGAA**gccgcctgcagcccgcgccccccgtgcccccgccccgccgccggcccgggcgcc** |
| rs2822654-rs1882882 | **gccgcctgcagcccgcgccccccgtgcccccgccccgccgccggcccgggcgcc**ACTAAGCCTTGGGGATCCAG | TGCTGTGGAAATACTAAAAGG |
| rs368657-rs376635 | CTCCAGAGGTAATCCTGTGA | **gccgcctgcagcccgcgccccccgtgcccccgccccgccgccggcccgggcgcc**TGGTGTGAGATGGTATCTAGG |
| rs2822731-rs2822732 | GTATAATCCATGAATCTTGTTT | **gccgcctgcagcccgcgccccccgtgcccccgccccgccgccggcccgggcgcc**TTCAAATTGTATATAAGAGAGT |
| rs1475881-rs7275487 | GCAGGAAAGTTATTTTTAAT | **gccgcctgcagcccgcgccccccgtgcccccgccccgccgccggcccgggcgcc**TGCTTGAGAAAGCTAACACTT |
| rs1735976-rs2827016 | **gccgcctgcagcccgcgccccccgtgcccccgccccgccgccggcccgggcgcc**CAGTGTTTGGAAATTGTCTG | GGCACTGGGAGATTATTGTA |
| rs447349-rs2824097 | **gccgcctgcagcccgcgccccccgtgcccccgccccgccgccggcccgggcgcc**TCCTGTTGTTAAGTACACAT | GGGCCGTAATTACTTTTG |
| rs418989- rs13047336 | ACTCAGTAGGCACTTTGTGTC | **gccgcctgcagcccgcgccccccgtgcccccgccccgccgccggcccgggcgcc**TCTTCCACCACACCAATC |
| rs987980- rs987981 | TGGCTTTTCAAAGGTAAAA | **gccgcctgcagcccgcgccccccgtgcccccgccccgccgccggcccgggcgcc**GCAACGTTAACATCTGAATTT |
| rs4143392- rs4143391 | TTGAAGAAAGGAGAATTTAA | **gccgcctgcagcccgcgccccccgtgcccccgccccgccgccggcccgggcgcc**ATTTTATATGTCATGATCTAAG |
| rs1691324- rs13050434 | **gccgcctgcagcccgcgccccccgtgcccccgccccgccgccggcccgggcgcc**AGAGATTACAGGTGTGAGC | ATGATCCTCAACTGCCTCT |
| rs11909758-rs9980111 | **gccgcctgcagcccgcgccccccgtgcccccgccccgccgccggcccgggcgcc**TGAAACTCAAAAGAGAAAAG | ACAGATTTCTACTTAAAATT |
| rs2826842-rs232414 | **gccgcctgcagcccgcgccccccgtgcccccgccccgccgccggcccgggcgcc**GCAAAGGGGTACTCTATGTA | TATCGGGTCATCTTGTTAAA |
| rs1980969-rs1980970 | TCTAACAAAGCTCTGTCCAAAA | **gccgcctgcagcccgcgccccccgtgcccccgccccgccgccggcccgggcgcc**CCACACTGAATAACTGGAACA |
| rs9978999-rs9979175 | **Gccgcctgcagcccgcgccccccgtgcccccgccccgccgccggcccgggcgcc**GCAAGCAAGCTCTCTACCTTC | TGTTCTTCCAAAATTCACATGC |
| rs1034346-rs12481852 | ATTTCACTATTCCTTCATTTT | **gccgcctgcagcccgcgccccccgtgcccccgccccgccgccggcccgggcgcc**TAATTGTTGCACACTAAATTAC |
| rs7509629-rs2828358 | **gccgcctgcagcccgcgccccccgtgcccccgccccgccgccggcccgggcgcc**ACTGTCATGGACTTAAACAA | TTCAGGAAAAAGTAATATGGAA |
| rs4817013-rs7277036 | **gccgcctgcagcccgcgccccccgtgcccccgccccgccgccggcccgggcgcc**AAAAAGCCACAGAAATCAGTC | TTCTTATATCTCACTGGGCATT |
| rs9981121-rs2829696 | **gccgcctgcagcccgcgccccccgtgcccccgccccgccgccggcccgggcgcc**GGATGGTAGAAGAGAAGAAAGG | TCACAAACATAAGAAATGGTGA |
| rs455921-rs2898102 | **gccgcctgcagcccgcgccccccgtgcccccgccccgccgccggcccgggcgcc**TGCAAAGATGCAGAACCAAC | TTTTGTTCCTTGTCCTGGCTGA |
| rs2898102- rs458848 | **gccgcctgcagcccgcgccccccgtgcccccgccccgccgccggcccgggcgcc**TGCAAAGATGCAGAACCAAC | GCCTCCAGCTCTATCCAAGTT |
| rs961301-rs2830208 | CCTTAATATCTTCCCATGTCCA | **gccgcctgcagcccgcgccccccgtgcccccgccccgccgccggcccgggcgcc**ATTGTTAGTGCCTCTTCTGCTT |
| rs2174536-rs458076 | **gccgcctgcagcccgcgccccccgtgcccccgccccgccgccggcccgggcgcc**GAGAAGTGAGGTCAGCAGCT | TTTCTAAATTTCCATTGAACAG |
| rs11088023-rs11088024 | **gccgcctgcagcccgcgccccccgtgcccccgccccgccgccggcccgggcgcc**GAAATTGGCAATCTGATTCT | CAACTTGTCCTTTATTGATGT |
| rs1011734-rs1011733 | CTATGTTGATAAAACATTGAAA | **gccgcctgcagcccgcgccccccgtgcccccgccccgccgccggcccgggcgcc**GCCTGTCTGGAATATAGTTT |
| rs2831244-rs9789838 | CAGGGCATATAATCTAAGCTGT | **gccgcctgcagcccgcgccccccgtgcccccgccccgccgccggcccgggcgcc**CAATGACTCTGAGTTGAGCAC |
| rs8132769-rs2831440 | **gccgcctgcagcccgcgccccccgtgcccccgccccgccgccggcccgggcgcc**AACTCTCTCCCTCCCCTCT | TATGGCCCCAAAACTATTCT |
| rs8134080-rs2831524 | ACAAGTACTGGGCAGATTGA | **Gccgcctgcagcccgcgccccccgtgcccccgccccgccgccggcccgggcgcc**GCCAGGTTTAGCTTTCAAGT |
| rs4817219-rs4817220 | **gccgcctgcagcccgcgccccccgtgcccccgccccgccgccggcccgggcgcc**TTTTATATCAGGAGAAACACTG | CCAGAATTTTGGAGGTTTAAT |
| rs2250911-rs2250997 | TGTCATTCCTCCTTTATCTCCA | **gccgcctgcagcccgcgccccccgtgcccccgccccgccgccggcccgggcgcc**TTCTTTTGCCTCTCCCAAAG |
| rs2831899-rs2831900 | ACCCTGGCACAGTGTTGACT | **gccgcctgcagcccgcgccccccgtgcccccgccccgccgccggcccgggcgcc**TGGGCCTGAGTTGAGAAGAT |
| rs2831902-rs2831903 | **gccgcctgcagcccgcgccccccgtgcccccgccccgccgccggcccgggcgcc**AATTTGTAAGTATGTGCAACG | TTTTTCCCATTTCCAACTCT |
| rs11088086-rs2251447 | **gccgcctgcagcccgcgccccccgtgcccccgccccgccgccggcccgggcgcc**AAAAGATGAGACAGGCAGGT | ACCCCTGTGAATCTCAAAAT |
| rs2832040-rs11088088 | **gccgcctgcagcccgcgccccccgtgcccccgccccgccgccggcccgggcgcc**GCACTTGCTTCTATTGTTTGT | CCCTTCCTCTCTTCCATTCT |
| rs2832141-rs2246777 | **gccgcctgcagcccgcgccccccgtgcccccgccccgccgccggcccgggcgcc**gtgggAGCACTGCAGGTA | ACAGATACCAAAGAACTGCAA |
| rs2832959 –rs9980934 | TGGACACCTTTCAACTTAGA | **Gccgcctgcagcccgcgccccccgtgcccccgccccgccgccggcccgggcgcc**gaacagtaatgttgaacttttt |
| rs2833734-rs2833735 | TCTTGCAAAAAGCTTAGCACA | **gccgcctgcagcccgcgccccccgtgcccccgccccgccgccggcccgggcgcc**AAAAAGATCTCAAAGGGTCCA |
| rs933121-rs933122 | **gccgcctgcagcccgcgccccccgtgcccccgccccgccgccggcccgggcgcc**GCTTTTGCTGAACATCAAGT | CCTTCCAGCAGCATAGTCT |
| rs2834140-rs12626953 | AAATCCAGGATGTGCAGT | **gccgcctgcagcccgcgccccccgtgcccccgccccgccgccggcccgggcgcc**ATGATGAGGTCAGTGGTGT |
| rs2834485-rs3453 | CATCACAGATCATAGTAAATGG | **gccgcctgcagcccgcgccccccgtgcccccgccccgccgccggcccgggcgcc**AATTATTATTTTGCAGGCAAT |
| rs9974986-rs2834703 | **gccgcctgcagcccgcgccccccgtgcccccgccccgccgccggcccgggcgcc**CATGAGGCAAACACCTTTCC | GCTGGACTCAGGATAAAGAACA |
| rs2776266-rs2835001 | **Gccgcctgcagcccgcgccccccgtgcccccgccccgccgccggcccgggcgcc**TGGAAGCCTGAGCTGACTAA | CCTTCTTTTCCCCCAGAATC |
| rs1984014-rs1984015 | **gccgcctgcagcccgcgccccccgtgcccccgccccgccgccggcccgggcgcc**TTAGGAGAACAGAAGATCAGAG | AAAGACTATTGCTAAATGCTTG |
| rs7281674-rs2835316 | TAAGCGTAGGGCTGTGTGTG | **gccgcctgcagcccgcgccccccgtgcccccgccccgccgccggcccgggcgcc**GGACGGATAGACTCCAGAAGG |
| rs13047304-rs13047322 | GAATGACCTTGGCACTTTTATCA | **Gccgcctgcagcccgcgccccccgtgcccccgccccgccgccggcccgggcgcc**AAGGATAGAGATATACAGATGAATGGA |
| rs2835545-rs4816551 | **gccgcctgcagcccgcgccccccgtgcccccgccccgccgccggcccgggcgcc**GGCCATGTTCTTGGAAGCTA | TTCACCTGAGTTGGGGAATG |
| rs2835735-rs2835736 | **gccgcctgcagcccgcgccccccgtgcccccgccccgccgccggcccgggcgcc**CATGCACCGCGCAAATAC | ATGCCTCACCCACAAACAC |
| rs13047608-rs2835826 | **gccgcctgcagcccgcgccccccgtgcccccgccccgccgccggcccgggcgcc**TCCAAGCCCTTCTCACTCAC | CTGGGACGGTGACATTTTCT |
| rs2836550-rs2212596 | **gccgcctgcagcccgcgccccccgtgcccccgccccgccgccggcccgggcgcc**CCCAGGAAGAGTGGAAAGATT | TTAGCTTGCATGTACCTGTGT |
| rs2836660-rs2836661 | AGCTAGATGGGGTGAATTTT | **gccgcctgcagcccgcgccccccgtgcccccgccccgccgccggcccgggcgcc**TGGGCTGAGGGGAGATTC |
| rs465612-rs8131220 | ATCAAGCTAATTAATGTTATCT | **Gccgcctgcagcccgcgccccccgtgcccccgccccgccgccggcccgggcgcc**AATGAATAAGGTCCTCAGAG |
| rs9980072-rs8130031 | TTTAATCTGATCATTGCCCTA | **gccgcctgcagcccgcgccccccgtgcccccgccccgccgccggcccgggcgcc**AGCTGTGGGTGACCTTGA |
| rs418359-rs2836926 | TGTCCCACCATTGTGTATTA | **Gccgcctgcagcccgcgccccccgtgcccccgccccgccgccggcccgggcgcc**TCAGACTTGAAGTCCAGGAT |
| rs7278447-rs7278858 | **gccgcctgcagcccgcgccccccgtgcccccgccccgccgccggcccgggcgcc**GCTTCAGGGGTGTTAGTTTT | CTTTGTGAAAAGTCGTCCAG |
| rs385787-rs367001 | CCATCATGGAAAGCATGG | **gccgcctgcagcccgcgccccccgtgcccccgccccgccgccggcccgggcgcc**TCATCTCCATGACTGCACTA |
| rs367001-rs386095 | GAGATGACGGAGTAGCTCAT | **gccgcctgcagcccgcgccccccgtgcccccgccccgccgccggcccgggcgcc**CCCAGCTGCACTGTCTAC |
| rs2837296-rs2837297 | TCTTGTTCCAATCACAGGAC | **gccgcctgcagcccgcgccccccgtgcccccgccccgccgccggcccgggcgcc**ATGCTGTTAGCTGAAGCTCT |
| rs2837381-rs4816672 | **gccgcctgcagcccgcgccccccgtgcccccgccccgccgccggcccgggcgcc**TGAAAGCTCCTAAAGCAGAG | TTGAAGAGATGTGCTATCAT |
| **^1^**dbSNP accession numbers of two tandem SNPs (separated by dash).  Bold fonts indicate the gc clamp. Primers without the “gc clamp” are labelled with 6FAM. | | |
