# Supplementary material for: Non-Invasive Prenatal Detection of Trisomy 21 Using Tandem Single Nucleotide Polymorphisms
Source: PLoS One. 2010 Oct 8;5(10):e13184. doi: 10.1371/journal.pone.0013184 (PMC2951898; doi:10.1371/journal.pone.0013184)
Supplement: Table S4 — Maternal plasma DNA analysis using Haplotype Ratio calculations. (0.06 MB DOC) [file pone.0013184.s008.doc]

**Table S4. Maternal plasma DNA analysis using Haplotype Ratio calculations.**

| **Sample #** | **Starting template (ng)** | **PCR**  **Method ^1^** | **Fetal (paternal) % in maternal plasma ^2^** | **HR^3^** | **95% confidence interval^4^** | **Fetal Karyotype** | **Chr21 call** | **Gestational week (weeks)** | **Maternal Age (years)** |
| --- | --- | --- | --- | --- | --- | --- | --- | --- | --- |
| FDT0601 | 3 | Direct PCR-Pfu | 7.38% | 1.21 | (1,1.65) | Normal | D21 | 9 | 35 |
| FDT0602 | 3 | Direct PCR-Pfu | 10.77% | 1.19 | (1.15,1.25) | T18 | D21 | 14.3 | 30 |
| **FDT0603** | **2** | **Direct PCR-Pfu** | **4.44%** | **2.29** | **(2.19,2.48)** | **T21** | **T21** | **35** | **26** |
| **FDT0604** | **3** | **Direct PCR-Pfu** | **1.60%** | **1.87** | **(1.67,2.29))** | **T21** | **T21** | **27.6** | **29** |
| **FDT0705** | **3** | **Direct PCR-Pfu** | **5.04%** | **2.09** | **(2.05,2.18)** | **T21** | **T21** | **25** | **42** |
| **FDT0706** | **5** | **MLA –Taq** | **3.29%** | **2.33** | **(2.19,2.62)** | **T21** | **T21** | **29.5** | **40** |
| **FDT0807** | **3** | **Direct PCR-Pfu** | **1.68%** | **2.29** | **(1.97,2.92)** | **T21** | **T21** | **31.3** | **33** |
| **FDT0808** | **3** | **Direct PCR-Pfu** | **7.12%** | **0.05** | **(0.03,0.11)** | **T21** | **T21** | **35.5** | **40** |
| FDT0809 | 3 | Direct PCR-Pfu | 5.46% | 1.29 | (1.07,1.72) | T18 | D21 | 12.7 | 43 |
| FDT0810 | 3 | Direct PCR-Pfu | 19.70% | 1.04 | (1.01,1.09) | Normal | D21 | 15 | 40 |
| FDT0812 | 5 | MLA –Taq | 8.00% | 1 | (0.98,1.06) | Normal | D21 | 12.6 | 38 |
| FDT0813 | 3 | MLA –Taq | 6.81% | 1 | (0.81,1.39) | Normal | D21 | 14.5 | 40 |
| FDT0816 | 4 | MLA –Taq | 7.32% | 0.9 | (0.62,1.47) | Normal | D21 | 26 | 25 |
| FDT0817 | 5 | MLA –Pfu | 24.05% | 1.04 | (1.01,1.1) | Normal | D21 | 13.5 | 30 |
| FDT0818 | 5 | MLA –Taq | 4.05% | 1.22 | (0.92,1.85) | Normal | D21 | 11.4 | 39 |
| FDT0821 | 3 | MLA –Taq | 2.93% | 1.06 | (1,1.19) | Normal | D21 | 11.4 | 28 |
| FDT0822 | 5 | MLA –Taq | 3.96% | 0.92 | (0.78,1.22) | Normal | D21 | 33.5 | 33 |
| FDT0824 | 5 | MLA –Pfu | 11.71% | 1.35 | (1.34,1.37) | Normal | D21 | 15.4 | 41 |
| FDT0827 | 4 | MLA –Taq | 9.20% | 0.95 | (0.88,1.09) | Normal | D21 | 30.2 | 32 |
| FDT0832 | 4 | MLA –Taq | 3.67% | 1.23 | (1.16,1.36) | Normal | D21 | 14.6 | 37 |
| FDT0833 | 4 | MLA –Taq | 7.00% | 1.46 | (1.42,1.53) | Normal | D21 | 36.1 | 24 |
| FDT0834 | 4 | MLA –Taq | 5.94% | 1.06 | (0.79,1.61) | Normal | D21 | 13.4 | 33 |
| **FDT0835** | **5** | **MLA –Taq** | **6.82%** | **2.08** | **(1.85,2.54)** | **T21** | **T21** | **20.3** | **22** |
| FDT0836 | 4 | MLA –Taq | 7.32% | 1.29 | (1.1,1.68) | Normal | D21 | 11.5 | 32 |
| FDT0837 | 5 | MLA –Pfu | 4.23% | 1.02 | (0.96,1.13) | Normal | D21 | 13.3 | 33 |
| FDT0839 | 5 | MLA –Taq | 13.74% | 0.9 | (0.83,0.92) | Normal | D21 | 19.6 | 29 |
| FDT0840 | 5 | MLA –Taq | 9.67% | 1.2 | (1.1,1.4) | Normal | D21 | 17 | 32 |
| HR, Haplotype Ratio; MLA-Taq, Multiplexed Linear Amplification using Taq polymerase; Direct PCR-Pfu, Direct PCR using Pfu polymerase; T21, Trisomy 21; D21, Disomy 21.  ^1^Direct PCR-Pfu indicates that Direct PCR or Sequence specific PCR was performed using high fidelity Pfu Ultra II fusion enzyme. MLA- Taq indicates that multiplexed linear amplification was performed using Hot star Taq enzyme followed by sequence specific PCR using Pfu Ultra II fusion enzyme. And MLA- Pfu indicates that MLA amplification was performed using Pfu Ultra II enzyme followed by sequence specific PCR also using Pfu Ultra II fusion enzyme.  ^2^Fetal % due to paternal contribution is calculated by using the formula listed under section “Tandem SNP analysis permits fetal chromosomal dosage determination in maternal plasma”.  ^3^Informative assay used to calculate the HR can be found in Table S2.  ^4^95% CI was calculated using the mean ± 1.96 SD/ √3  Bold fonts indicate the fetal T21 subjects/samples. | | | | | | | |  |  |
