# Supplementary material for: Non-Invasive Prenatal Detection of Trisomy 21 Using Tandem Single Nucleotide Polymorphisms
Source: PLoS One. 2010 Oct 8;5(10):e13184. doi: 10.1371/journal.pone.0013184 (PMC2951898; doi:10.1371/journal.pone.0013184)
Supplement: Table S5 — Informative tandem SNP assays and subject number. (0.03 MB DOC) [file pone.0013184.s009.doc]

Table S5. Informative tandem SNP assays and subject number.

| **Informative tandem SNP assay # ^1^** | **Sample/Subject number** |
| --- | --- |
| rs1735976-rs2827016 | FDT0601, FDT0808, FDT0813, FDT0821, FDT0840 |
| rs418989-rs13047336 | FDT0602, FDT0603, FDT0807, FDT0809, FDT0810, FDT0824 |
| rs11088086-rs2251447 | FDT0705, FDT0833 |
| rs8134080-rs2831524 | FDT0604 |
| rs2832141-rs2246777 | FDT0706, FDT0812, FDT0822, FDT0834 |
| rs2834485-rs3453 | FDT0816, FDT0817, FDT0827, FDT0836 |
| rs2822731-rs2822732 | FDT0818 |
| rs961301-rs2830208 | FDT0832 |
| rs418359-rs2836926 | FDT0835, FDT0839 |
| rs13047304-rs13047322 | FDT0837 |
| **^1^**dbSNP accession numbers of two tandem SNPs (separated by dash). | |
