# Supplementary material for: Non-Invasive Prenatal Detection of Trisomy 21 Using Tandem Single Nucleotide Polymorphisms
Source: PLoS One. 2010 Oct 8;5(10):e13184. doi: 10.1371/journal.pone.0013184 (PMC2951898; doi:10.1371/journal.pone.0013184)
Supplement: Table S6 — MLA followed by CTCE analysis. Starting from approximately 9 copies per tube (Mix 1a, 1b, 1c) following MLA, approximately 47 copies were created per cycle or a yield greater than 500%. (0.06 MB DOC) [file pone.0013184.s010.doc]

Table S6. MLA followed by CTCE analysis. Starting from approximately 9 copies per tube (Mix 1a, 1b, 1c) following MLA, approximately 47 copies were created per cycle or a yield greater than 500%.

| **Before MLA^1^** | **Area under Wild Type peak (volt-seconds)** | **Area under Mutant peak (volt-seconds)** | **(WT): (MT)** | **WT (copies)** |
| --- | --- | --- | --- | --- |
| Mix 1a (1ul)+10 copies of IS | 291 | 376 | 0.77 : 1 | 7.7 |
| Mix 1a (1ul)+10 copies of IS | 24,410 | 69,518 | 0.35 :1 | 3.5 |
| Mix 1a (1ul)+10 copies of IS | 18,579 | 27,309 | 0.68 :1 | 6.8 |
| Mix 1b (1ul)+10 copies of IS | 1,931 | 2,400 | 0.8 :1 | 8 |
| Mix 1b (1ul)+10 copies of IS | 835 | 591 | 1.4 :1 | 14 |
| Mix 1b (1ul)+10 copies of IS | 2,013 | 5,533 | 0.36 :1 | 3.6 |
| Mix 1c (1ul)+10 copies of IS | 7,579 | 15,656 | 0.48 :1 | 4.8 |
| Mix 1c (1ul)+10 copies of IS | 2,201 | 1,632 | 1.34 :1 | 13.4 |
| Mix 1c (1ul)+10 copies of IS | 2,874 | 1,479 | 1.94 :1 | 19.4 |
|  |  |  | Average (WT:MT copies) | = 9.02 :10 |
|  |  |  |  |  |
| **After MLA** |  |  |  |  |
| MLA mix 2a (1ul)+1,000 copies of IS | 1,23,217.5 | 1,15,683.5 | 1.06 :1 | 1,060 |
| MLA mix 2a (1ul)+1,000 copies of IS | 26,127.50 | 20,454.50 | 1.3 :1 | 1,300 |
| MLA mix 2a (1ul)+1,000 copies of IS | 17,829 | 10,689 | 1.7 :1 | 1,700 |
| MLA mix 2b (1ul)+1,000 copies of IS | 1,89,877.5 | 3,74,70.5 | 5.07 :1 | 5,070 |
| MLA mix 2b (1ul)+1,000 copies of IS | 1,35,500 | 27,385 | 4.94 :1 | 4,940 |
| MLA mix 2b (1ul)+1,000 copies of IS | 1,14,898 | 27,681 | 4.15:1 | 4,150 |
| MLA mix 2c (1ul)+1,000 copies of IS | 23,500 | 85,461 | 0.27 :1 | 270 |
| MLA mix 2c (1ul)+1,000 copies of IS | 37,722.50 | 108202.5 | 0.34 :1 | 340 |
| MLA mix 2c (1ul)+1,000 copies of IS | 1,144 | 2,640 | 0.43 :1 | 430 |
|  |  |  | Average (WT:MT copies) | = 2,140:1,000 |
|  |  |  | Amplification | = 47.35 copies per cycle |
|  |  |  | Yield | = 524.9% |
| WT, Wild type; MT, Mutant; WT copies, Wild Type copies; MLA, Multiplexed Linear Amplification.  **^1^**Mix 1a (1ul) +10 copies of IS, indicate that the template is 1ul from "Mix 1a" tube plus volume (equivalent to 10 copies) of internal standard (IS) sequences. Similarly the others.  Before MLA, wild type copies are approximately 9. There is an amplification of 47.35 copies per cycle due MLA resulting in a yield of 524.9%. | | | | |
