# Supplementary material for: Non-Invasive Prenatal Detection of Trisomy 21 Using Tandem Single Nucleotide Polymorphisms
Source: PLoS One. 2010 Oct 8;5(10):e13184. doi: 10.1371/journal.pone.0013184 (PMC2951898; doi:10.1371/journal.pone.0013184)
Supplement: Text S1 — Supporting Information. (0.07 MB DOC) [file pone.0013184.s011.doc]

Supporting Information

**Sample processing and DNA quantification**

12-20 mls of peripheral blood drawn from each subject was collected in purple top (EDTA) tubes and centrifuged at 200 x g for 10 minutes with no acceleration and no brake, supernatant was transferred to a 15 ml conical and centrifuged at 1,600 x g for 10 minutes with no acceleration and no brake, plasma was transferred to low binding micro centrifuge tubes and centrifuged at 16,000 x g for 1 min. Cell-free plasma was stored in 2 ml low binding tubes at -80ºC until DNA was further processed. DNA was extracted from cell-free plasma using the QIAamp UltraSens Kit (Qiagen, Valencia, California) according to manufacturer’s instructions with the first centrifugation optimized at 700 x g. Genomic DNA (gDNA) extraction of the buffy coat was done with the Gentra Puregene Blood Kit Plus (Qiagen Inc., Valencia, California) according to the manufacturer’s instructions. gDNA was quantified by quantitative real-time PCR using TaqMan. One sample was lost during DNA extraction (FDT0823) and six samples did not have enough DNA to proceed with analysis due to low recovery (<2 ng), these were (FDT0814 (12 ml), FDT0815 (12 ml), FDT0828 (12 ml), FDT0829 (12 ml), FDT0831 (12 ml), FDT0838 (16 ml). Six samples were not informative at the 9-16 tandem SNP assays tested before running out of sample (FDT0811, FDT0819, FDT0820, FDT0825, FDT0826, FDT0830) (**Table S2**).

**Quantitative real-time PCR**

Concentrations of maternal buccal swab and plasma/serum samples were determined by quantitative real-time PCR using TaqMan chemistry. TaqMan primers (Applied Biosystems, Foster City, California) and probes (Applied Biosystems, Foster City, California) for a homozygous site on chr19 were designed using the Primer Express v2.0 software (Applied Biosystems, Foster City, California) following the software recommendations. The forward primer sequence was 5’-*GTTCCCGTAACTGAAACATCAATG*-3’(Tm=58ºC, G-C content=42%, Length=24 bp), reverse primer sequence was 5’-*AAGGAACCTGAGGATGCT GTGT*-3’ (Tm=59ºC, G-C content=50%, Length=22 bp) and probe sequence was 5’-*CAGGCTC AGCTGGT*-3’(Tm=67.3ºC, 14 bp long, G-C content=64.3% and NED labelled) (82 bp product). We performed a standard curve by serial dilutions of the TK6 cell line gDNA (standard). Standard curve for quantitative real-time PCR was setup with final concentrations of 1X TaqMan Master mix (Applied Biosystems, Foster City, California), 200 nM probe, 900 nM forward primer and reverse primer (each), TK6 DNA (standard) or 1μl of maternal sample (unknown) and nuclease free water in a final reaction volume of 10 μl. Reactions were set up in triplicate. The loaded 384 well plate (Eppendorf, Westbury, New York) was covered with an optical adhesive film (Applied Biosystems, Foster City, California) and centrifuged for 2 minutes at 2,500 rpm. We then used 7900HT Fast real-time PCR system (Applied Biosystems, Foster City, California) to run the quantitative real-time PCR under the cycling conditions of 50^0^C for 2 min followed by 95^0^C for 10 min, then 40 cycles of 92^0^C for 15 sec and 60^0^C for 1 min. The quantity/concentration of unknown maternal sample was obtained by using Sequence Detection System (SDS) software V2.3 (Applied Biosystems, Foster City, California). TK6 genomic DNA standards were used for standard curve analysis. Although the TK6 cell line is stable and maintains its karyotype in culture [1], but because TK6 genomic DNA was extracted while cells were in exponential growth, an adjustment factor of 2.5 is required to estimate gDNA amounts for samples derived from blood at the chromosome 19 locus (lab observation).

**MLA development and optimization**

gDNA was isolated from the TK6 cell line, a diploid human male lymphoblast line [1], and used as template DNA. 12 target sequences were linearly amplified (forward primers) using 12 oligonucleotide primers. The oligonucleotides (synthesized by Sigma Genosys) all had a 54-mer GC rich 5’ tail (*gccgcctgcagcccgcgccccccgtgcccccgccccgccgccggcccgggcgcc*) 5’ to an approximately 20-mer target-specific region [2]. These were added to a master mix with a combined total primer concentration equal to 0.6 μM. The master mix had final concentrations of 1X Buffer with 15 mM MgCl2 (Qiagen Inc., Valencia, California), 0.125 mM dNTPs (Fermentas Inc., Geln Burnie, Maryland), 0.04 Units/μl of HotStar Taq DNA polymerase (Qiagen Inc., Valencia, California) or 1X Buffer (Qiagen Inc., Valencia, California), 0.1 mM dNTPs (Fermentas Inc., Geln Burnie, Maryland), 100 ng/μl BSA (New England Biolabs, Ipswich, Massachusetts), 2 μl of PfuUltra II Fusion HS DNA polymerase (Qiagen Inc., Valencia, California) and 0.2μM GC-rich primers (Sigma Genosys, The Woodlands, Texas) and nuclease free water in a final reaction volume of 50 μl. Ten primers targeted chromosome 21 tandem SNPs, one targeted a region on chromosome 19, and one targeted the human renin gene (forward primer sequence was 5’- *ACCCCTTCTCTGCCTGAGTT* -3’ and reverse primer sequence with GC clamp was 5’ -*gccgcctgcagcccgcgccccccgtgcccccgccccgccgccggcccgggcgccGCCACA GCCACAGAACCTCAGTGGAT* -3’), 2 ng of gDNA (TK6) was used as starting template in each replicate sample. Three tubes were set aside as the “before MLA” set (no Taq was added to these three tubes) and three tubes were set aside as the “after MLA” (see **Figure 5**). A negative control used nuclease free water as template. Thermal cycler conditions (Mastercycler ep gradientS (Eppendorf, Westbury, New York)) were as follows: 95^0^ for 15 min, 45 cycles at 95^0^ for 30 sec, annealing at 50^0^ for 30 sec, extension at 72^0^ for 45 sec and then the final extension at 72^0^ for 10 min.

To determine the efficiency of MLA, the copy number of the target sequences in the before MLA and after MLA tubes were measured. The human renin gene (*REN*) assay was quantified using competitive PCR where a known amount of an artificial mutant PCR product containing a single base pair difference from the wild-type was added in to the renin gene sequence-specific PCR along with 1 μl of the “before MLA” or 1 μl of the “after MLA” samples.

Final concentrations for the sequence specific PCR were 1X Buffer (Qiagen Inc., Valencia, California), 0.1 mM dNTPs (Fermentas Inc., Geln Burnie, Maryland), 100 ng/μl BSA (New England Biolabs, Ipswich, Massachusetts), 2 μl of PfuUltra II Fusion HS DNA polymerase (Qiagen Inc., Valencia, California) and 0.2 μM sequence specific (Sigma Genosys, The Woodlands, Texas). The mutant sequences were added in at 10 copies, 100 copies and 1,000 copies per reaction to enable quantification of DNA copies before and after MLA. The sequence-specific PCR was subjected to the following cycling conditions: 95^0^ for 15 min; 42 cycles of 95^0^ for 30 sec, annealing at 50^0^ for 30 sec, extension at 72^0^ for 45 sec and then the final extension at 72^0^ for 10 min.

Cycling Temperature Capillary Electrophoresis (CTCE) analysis was then performed on the PCR products using the MegaBACE^TM^, a 96-capillary DNA sequencer (GE Healthcare Bio-Sciences Corp, Piscataway, New Jersey) (see Methods for CTCE data analysis). Analysis from CTCE demonstrated yields greater than 100% at the renin gene locus when multiplexed with 12 GC-rich primers (see **Figure 5** and **Table S6**).

**Ligation-mediated PCR**

  gDNA was randomly fragmented by nebulization in 1X TE at 30 PSI.  Nebulized gDNA was polished in a reaction mixture of 1X Buffer 2 (New England Biolabs, Ipswich, Massachusetts), 83 ng/μl BSA (New England Biolabs, Ipswich, Massachusetts),  1mM dNTPs (Fermentas Inc., Geln Burnie, Maryland), 0.83 mM ATP (Roche, Indianapolis, IN), 0.15 U/μl T4 DNA polymerase (New England Biolabs, Ipswich, Massachusetts),  and 0.5 U/μl T4 Polynucleotide Kinase (New England Biolabs, Ipswich, Massachusetts) and incubated for 20 minutes at 12^0^C, 20 minutes at 25^0^C, and 20 minutes at 75^0^C in a Mastercycler ep gradientS (Eppendorf, Westbury, New York) thermal cycler.  The linkers were ligated to the blunt ended DNA in a reaction mixture of 1.2 μM linkers (gSEL3 and gSEL4) (Roche, Indianapolis, IN), 0.4X  Buffer 2 (New England Biolabs, Ipswich, Massachusetts), 2 mM ATP (Roche, Indianapolis, IN), and 100 U/μl T4 DNA Ligase (New England Biolabs, Ipswich, Massachusetts),  and incubated at 25^0^C for 90 minutes in a Mastercycler ep gradientS (Eppendorf, Westbury, New York) thermal cycler.  The linker ligated fragments (4 ng of DNA) were then amplified in a reaction of 1X Pfu Ultra II Fusion HS DNA polymerase buffer (Stratagene, La Jolla, California), 100 ng/μl BSA (New England Biolabs, Ipswich, Massachusetts), 0.1 mM dNTPs (Fermentas Inc., Geln Burnie, Maryland),  2 μM oligo (either LMPRC3 (Roche, Indianapolis, IN), or 297 – (*gccgcctgcagcccgcgccccccgtgcccccgccccgccgccggcccgggcgccCUCGAGAAUUCUGGAUCCUC* ) (Sigma Genosys, The Woodlands, Texas), a 1:25 dilution of Pfu Ultra II Fusion HS DNA polymerase (Stratagene, La Jolla, California), and 0.001 U/μl **PfuTurbo Hotstart DNA Polymerase in a** Mastercycler ep gradientS (Eppendorf, Westbury, New York) thermal cycler. Cycling conditions were 95^0^ for 2 min followed by denaturation at 95^0^ for 30 sec, 42 cycles at 95^0^ for 60 sec, annealing at 60^0^ for 30 sec, extension at 72^0^ for 10 sec and then the final extension at 72^0^ for 30 sec.  The LM-PCR product was used as a template in setting up the “sequence-specific” PCR as a second step. Sequence-specific PCR was set up as a high-fidelity reaction using 3 ul of LM-PCR product as template (see section High-Fidelity PCR and CTCE conditions for details).

**Tandem SNP PCR/CTCE assay design**

PCR assays for tandem SNP target sequences were designed using Primer 3.0 (Whitehead Institute, Cambridge, Massachusetts) and Winmelt software (Medprobe, Oslo, Norway) to determine theoretical melting profiles of these sequences. One of the primers flanking the target sequence is approximately 20 bases in length and labelled with a 5’6-FAM. The other primer is approximately 74 bases including an approximately 20-base target-specific sequence and a 54-base GC-rich clamp sequence [2]. Primer sets were ordered from (Sigma Genosys, The Woodlands, Texas). The oligonucleotide sequences for 58 assays are listed under **Table S3.**

A standard HiFi PCR condition was applied to all target sequences directly or after MLA, varying only annealing temperatures. These PCR amplicons were subjected to CTCE electrophoretic separation. The resulting electropherogram was analyzed for yield and purity of the PCR products. The purity was evaluated by comparing the peak area of the desired products to that of the by-products and nonspecific amplification. Target sequences which could be amplified with a high PCR efficiency (≥ 45% per cycle) and low levels of by-products and nonspecific amplification (≤ 0.1% of the desired products) went on to CTCE optimization.

**Eliminating the heteroduplexes**

The presence of heteroduplex molecules complicate the quantification and interpretation of haplotype analysis, therefore we adopted the method of “reconditioning PCR” to convert all targets to homoduplexes prior to CTCE injection [3]. Briefly, each reaction was set up with final concentrations of 1X PfuUltra II Fusion HS DNA polymerase buffer (Stratagene, La Jolla, California), 0.1 mM dNTPs (Fermentas Inc., Geln Burnie, Maryland), 0.8 μl of the PfuUltra II Fusion HS DNA polymerase (Stratagene, La Jolla, California), 100 ng/μl BSA (New England Biolabs, Ipswich, Massachusetts), 0.2 μM of each primer (Sigma Genosys, The Woodlands, Texas), 1 μl of 10-fold diluted PCR product and 14 μl of nuclease free water in a final reaction volume of 20 μl. The “reconditioning PCR” was then performed using the Mastercycler ep gradientS (Eppendorf, Westbury, New York) thermal cycler under the cycling conditions of 95^0^ for 2 min followed by 3 cycles at 95^0^ for 30 sec, 55^0^ for 30 sec, 72^0^ for 30 sec and then 72^0^ for 10 min.

**Buccal swab adjustment**

The ratio of peak 1 (p1) to peak 2 (p2) in heterozygous maternal buccal swabs is theoretically expected to be one. However, some target sequences have characteristic profiles where p1 > p2 or p2 > p1. When the average difference between p1 and p2 peaks exceeded 100 units (nine cases), the plasma peak areas were adjusted. In cases where p1 > p2 we used the formula

$Adjusted[p1(plasma)]=p1(plasma)*[(p2/p1)(maternalbuccalswab)]$ (1)

and in cases where p2 > p1 the formula used was

$Adjusted[p2(plasma)]=p2(plasma)*[(p1/p2)(maternalbuccalswab)]$ (2)

where p1 is the area under peak 1 and p2 is the area under peak 2 in the CTCE electropherogram. This adjustment (normalization), did not affect D21 or T21 calls but is expected to have an effect on a population-based ROC cut point, and is therefore highly recommended.

**Haplotype frequency estimation of tandem SNP regions**

Haplotype frequency estimation for HapMap Phase II+III data was carried out using Haploview version 4.2 [4]. Custom blocks were created for each tandem region and all regions were analyzed in batch. HapMap populations were analyzed independently, to generate haplotype frequency distributions per population. A minimum genotyping proportion of 0.3 was needed for a marker to be included in the haplotype estimates.

**Reconstruction experiments**

To simulate DNA isolated from maternal plasma and test the analytical precision of the overall process, mixtures containing known amounts of DNA isolated from the cord blood of a karyotyped baby and DNA isolated from the white blood cells of the mother were prepared. These studies were approved by IRB #1 in adherence to MCW study number PRO00009733. Because the fetal fraction of DNA in plasma varies *in vivo* [5,6] we combined the maternal and fetal DNA in a range of physiologically relevant concentrations. The simulated concentrations were aimed to mimic fetal DNA at 2.5%, 5%, 10%, 20%, and 40% for both a confirmed T21 infant and mother and a euploid infant and mother, although these estimates were based off of initial picogreen readings. True paternal percentages were approximately in the range of 2.4-4% for the 2.5% mixture, 3.7-7% for the 5% mixture, 3-9% for the 10% mixture, 6-14% for the 20% mixture and 17-24% for the 40% mixture when quantified by CTCE. **Figure S3** shows representative electropherograms from the T21 infant-maternal mix. For each analysis, we used 4 ng as starting template DNA from each mixture.

**Comparison of MLA to Direct PCR**

To determine HRs obtained following MLA (described in Methods) in comparison to the direct PCR protocol, another reconstruction experiment was performed on both T21 mixtures and euploid mixtures starting from 4 ng DNA template from the above mixtures. As seen in the boxplot of **Figure S4,** these data demonstrate that HRs calculated following MLA are not significantly different from HRs obtained following Direct PCR. Tandem SNP target encompassing SNPs rs1735976-rs2827016 was used for the euploid infant-maternal mixture, and SNPs rs2832141-rs2246777 was used for the T21 infant-maternal mixture.

**References**

1. Skopek TR, Liber HL, Penman BW, Thilly WG (1978) Isolation of a human lymphoblastoid line heterozygous at the thymidine kinase locus: possibility for a rapid human cell mutation assay. Biochem Biophys Res Commun 84: 411-416.

2. Cariello NF, Keohavong P, Kat AG, Thilly WG (1990) Molecular analysis of complex human cell populations: mutational spectra of MNNG and ICR-191. Mutat Res 231: 165-176.

3. Thompson JR, Marcelino LA, Polz MF (2002) Heteroduplexes in mixed-template amplifications: formation, consequence and elimination by 'reconditioning PCR'. Nucleic Acids Res 30: 2083-2088.

4. Barrett JC, Fry B, Maller J, Daly MJ (2005) Haploview: analysis and visualization of LD and haplotype maps. Bioinformatics 21: 263-265.

5. Lo YM, Tein MS, Lau TK, Haines CJ, Leung TN, et al. (1998) Quantitative analysis of fetal DNA in maternal plasma and serum: implications for noninvasive prenatal diagnosis. Am J Hum Genet 62: 768-775.

6. Lun FM, Chiu RW, Allen Chan KC, Yeung Leung T, Kin Lau T, et al. (2008) Microfluidics digital PCR reveals a higher than expected fraction of fetal DNA in maternal plasma. Clin Chem 54: 1664-1672.
